# Supplementary material for: NH Stretching Frequencies of Intramolecularly Hydrogen-Bonded Systems: An Experimental and Theoretical Study
Source: Molecules. 2021 Dec 17;26(24):7651. doi: 10.3390/molecules26247651 (PMC8706864; doi:10.3390/molecules26247651)
Supplement: Supplementary file 1 [file molecules-26-07651-s001.zip › molecules-1483947-supplementary.pdf]

NH stretching frequencies of intramolecularly hydrogen bonded systems. An experimental and theoretical study.

Poul Erik Hansen,[a] Mohammad Vakili,[b] Fadhil S. Kamounah,[c] and Jens Spanget-Larsen[a]

a. Department of Science and Environment, Roskilde University, Universitetsvej 1, DK-4000 Roskilde, Denmark

b. Department of Chemistry, Faculty of Science, Ferdowsi University of Mashhad, Mashhad, Iran.

c. Department of Chemistry, Universitetsparken 5, University of Copenhagen, Denmark

Supplementary Material

S1. IR spectrum of compound **6**.

S2. IR spectrum of compound **11**.

S3. IR spectrum of compound **17** (see also **S15)**.

S4. IR spectrum of compound **19**.

S5. IR spectrum of compound **45**.

S6. IR spectrum of compound **14**.

S7. IR spectrum of compound **16**.

S8. IR spectrum of compound **44**.

S9. IR spectrum of compound **8**.

S10. IR spectrum of compound **9**.

S11. IR spectrum of compound **10**.

S12. Experimental NH stretching wavenumbers vs. those calculated within the harmonic approximation with B3LYP/6-31G(d).

S13. Harmonic NH stretching wavenumbers computed with B3LYP/6-311++G(d,p) vs. those obtained with B3LYP/6-31G(d).

S14. B3LYP/6-31G(d) anharmonic VPT2 frequencies vs. the corresponding harmonic ones.

S15. Subtraction IR spectra of compound **17.**

S16. Harmonic NH stretching wavenumbers vs.NH bond distances (B3LYP/6-31G(d)).

S17. Observed NH stretching wavenumbers vs. hydrogen bond energies, E_{HB}, estimated according to Espinosa et al. - Linear fit.

S18. Observed NH stretching wavenumbers vs. hydrogen bond energies, E_{HB}, estimated according to Espinosa et al. - Parabolic fit.

S19. Optimized molecular structures of **17** computed with B3LYP/6-311++G(d,p) with relative energies in kcal/mol.

S20. Fundamental band assignments for **17** (17-H) and deuterated **17** (17-D).

S21. Materials section: Synthesis, characterization, and NMR spectra.

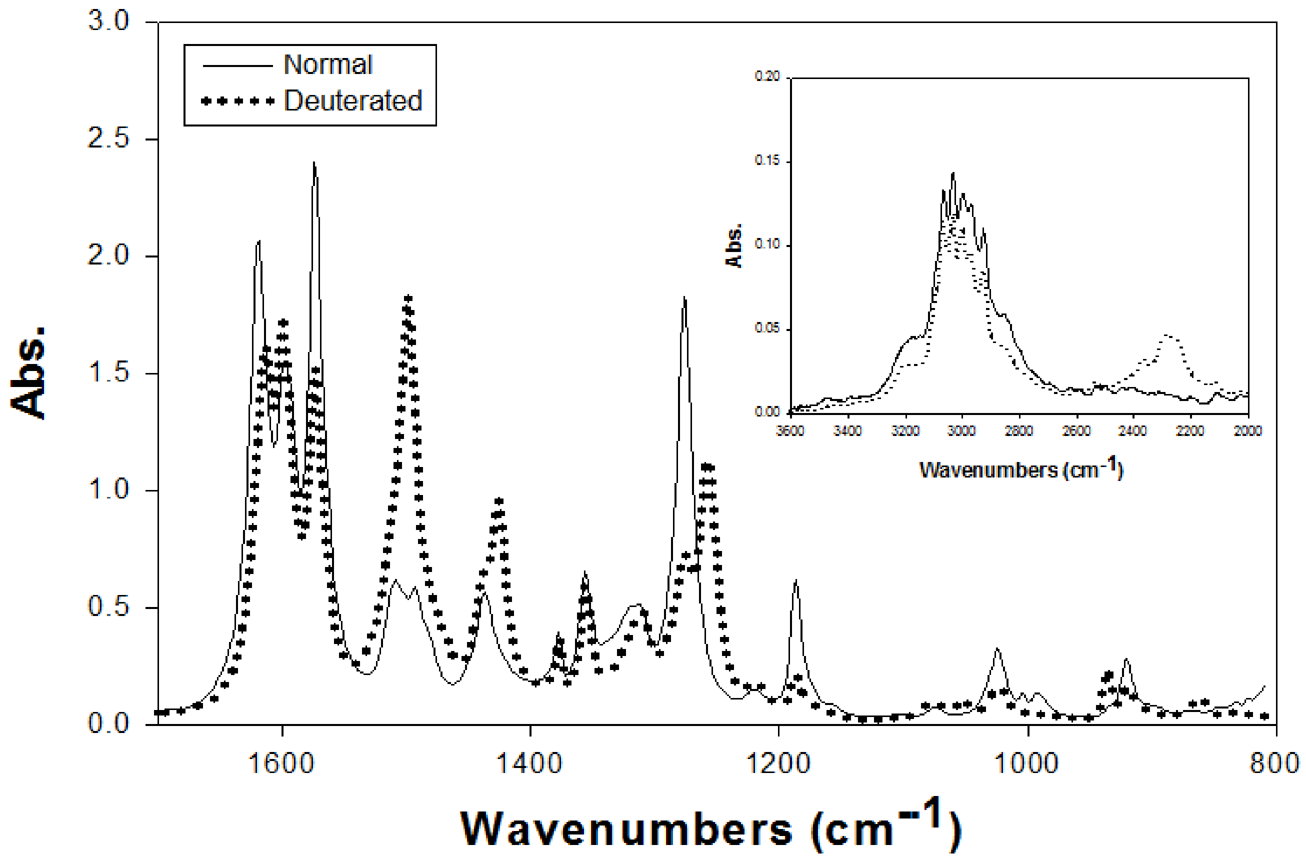

S1. IR spectrum of compound **6**.

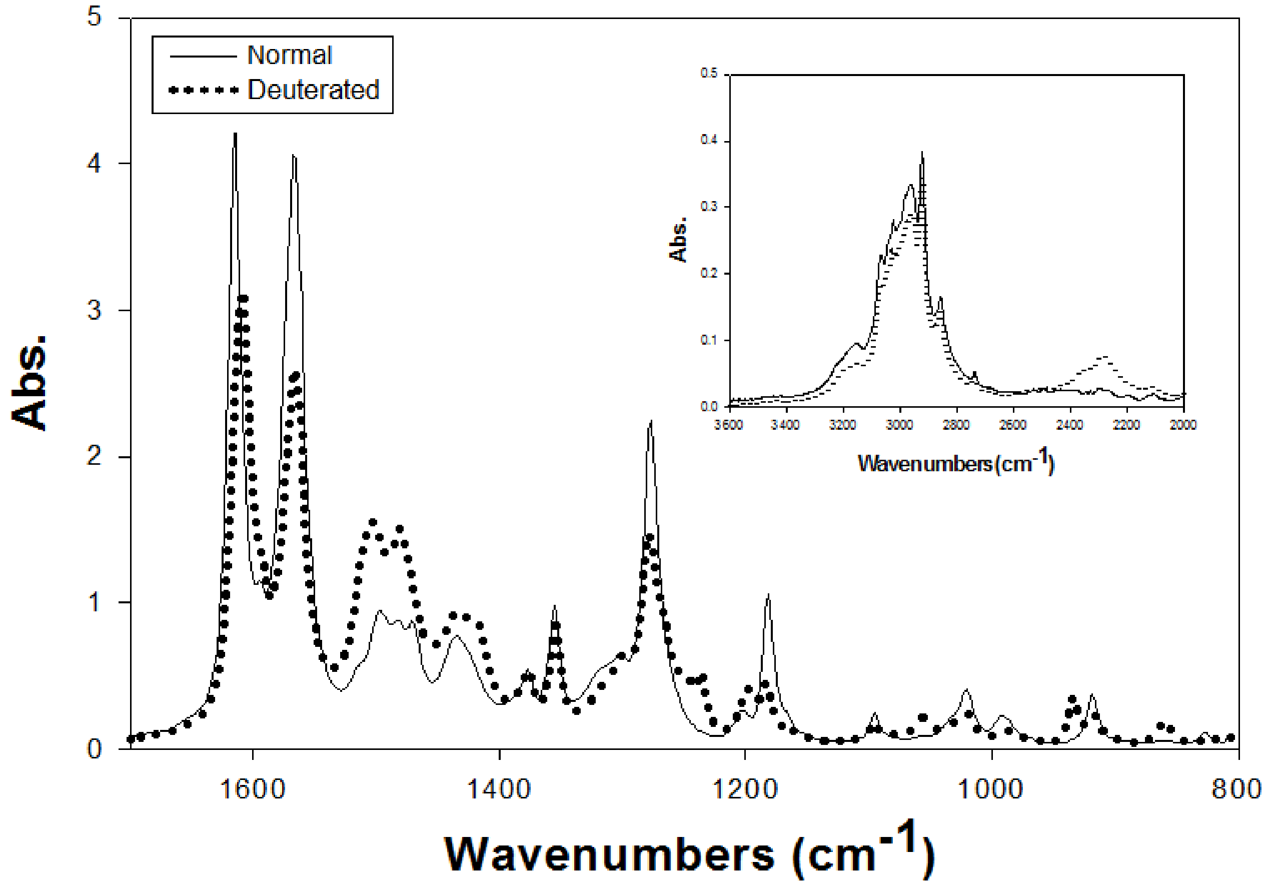

S2. IR spectrum of compound **11**.

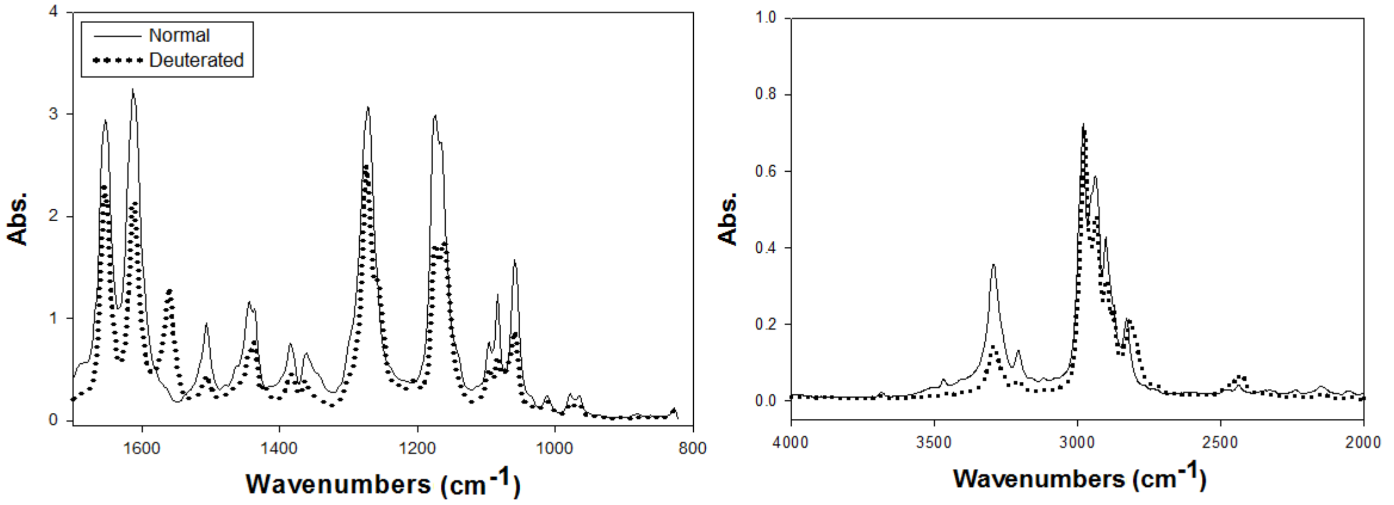

S3. IR spectrum of compound **17** (see also **S15)**.

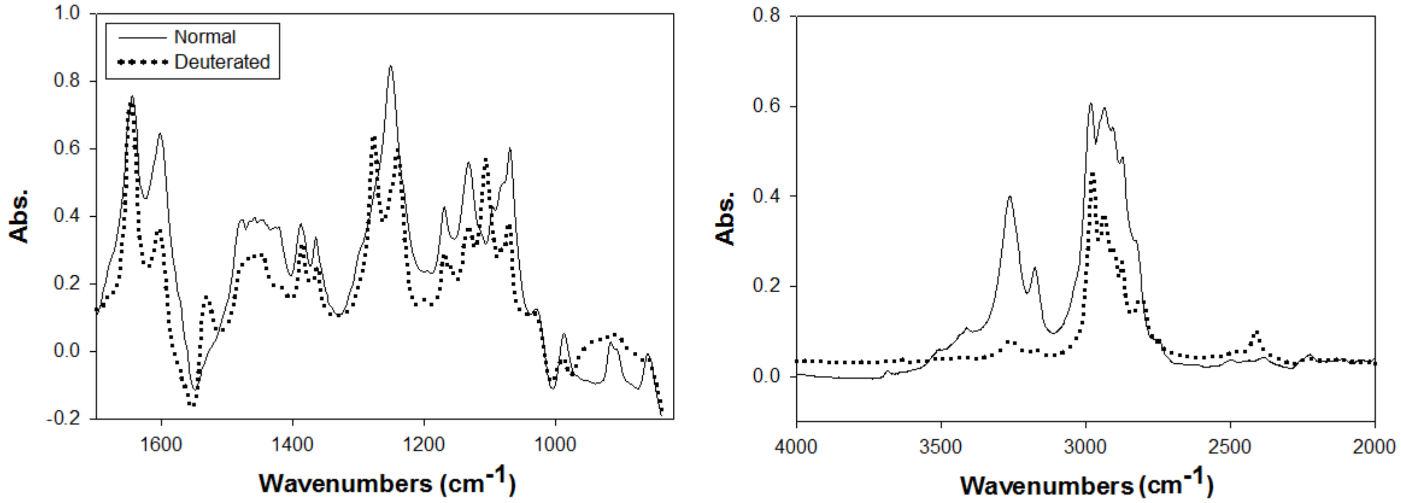

S4. IR spectrum of compound **19**.

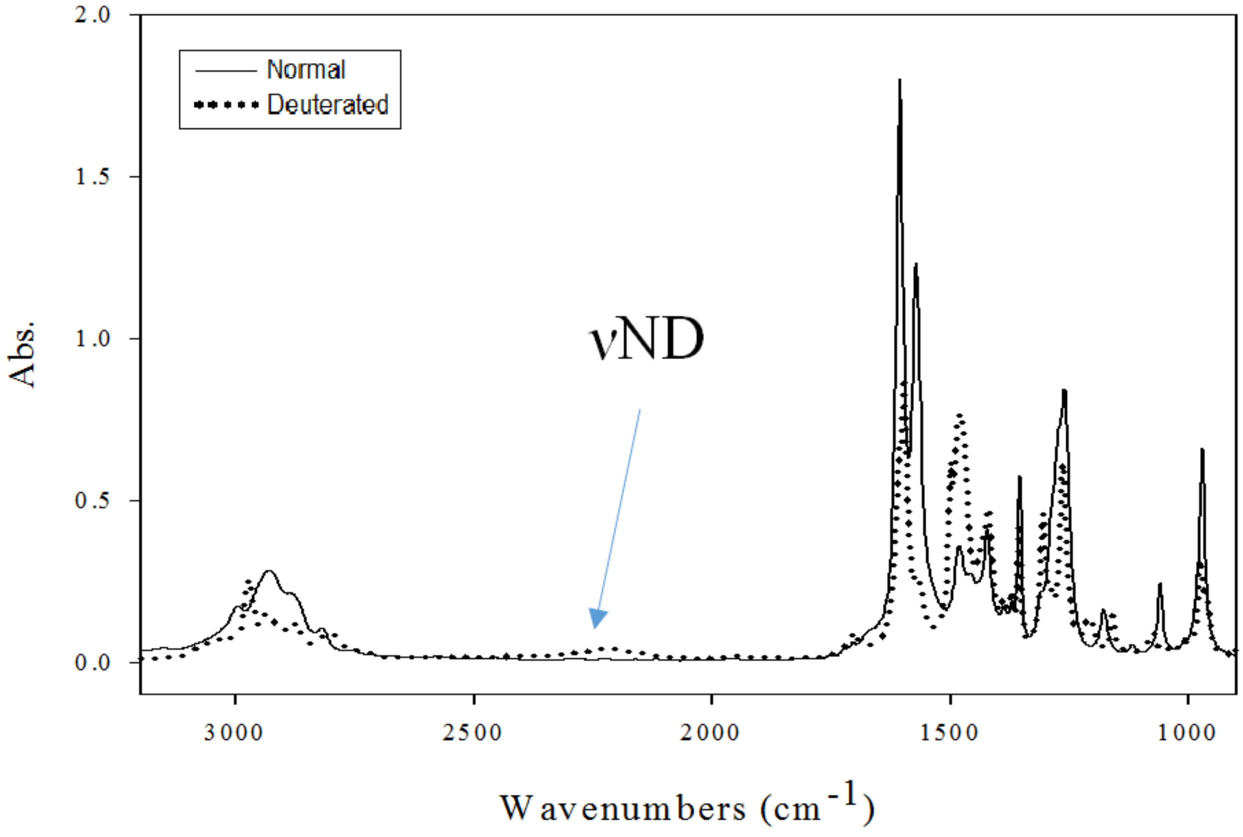

S5. IR spectrum of compound **45.**

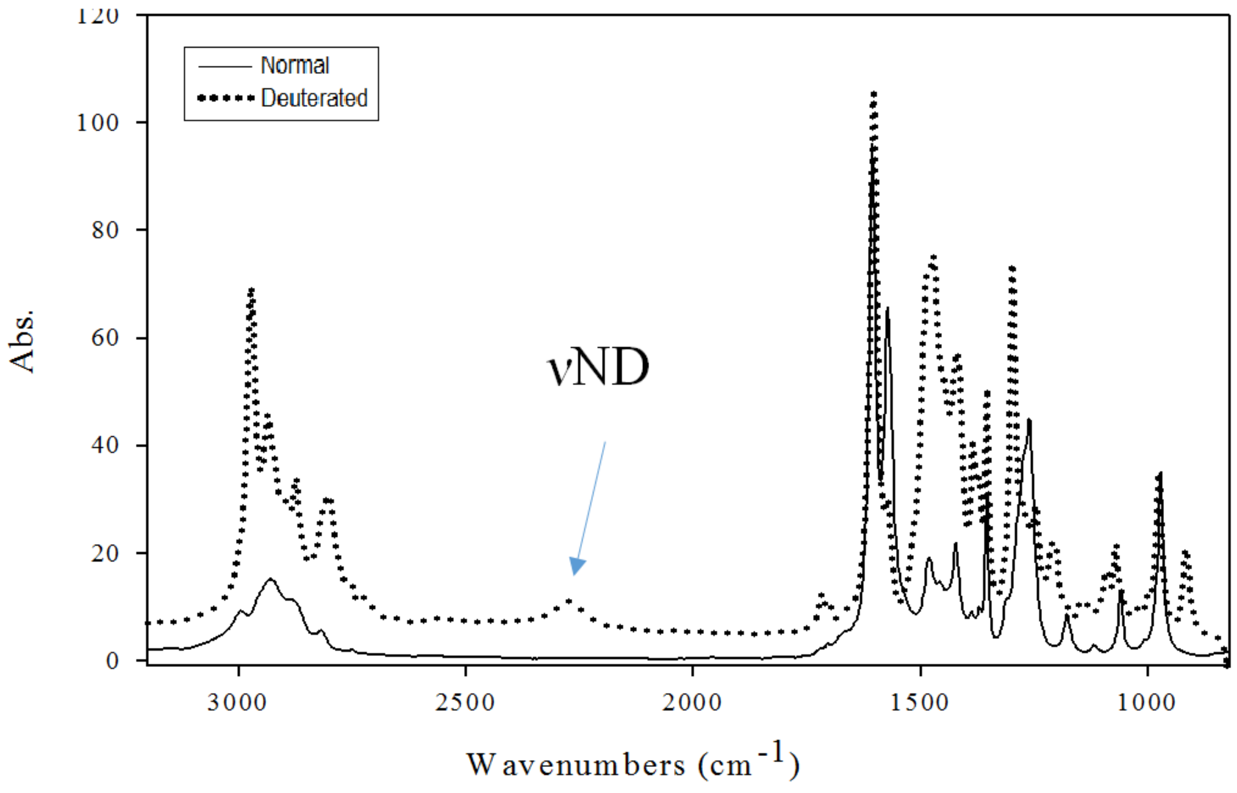

S6. IR spectrum of compound **14**.

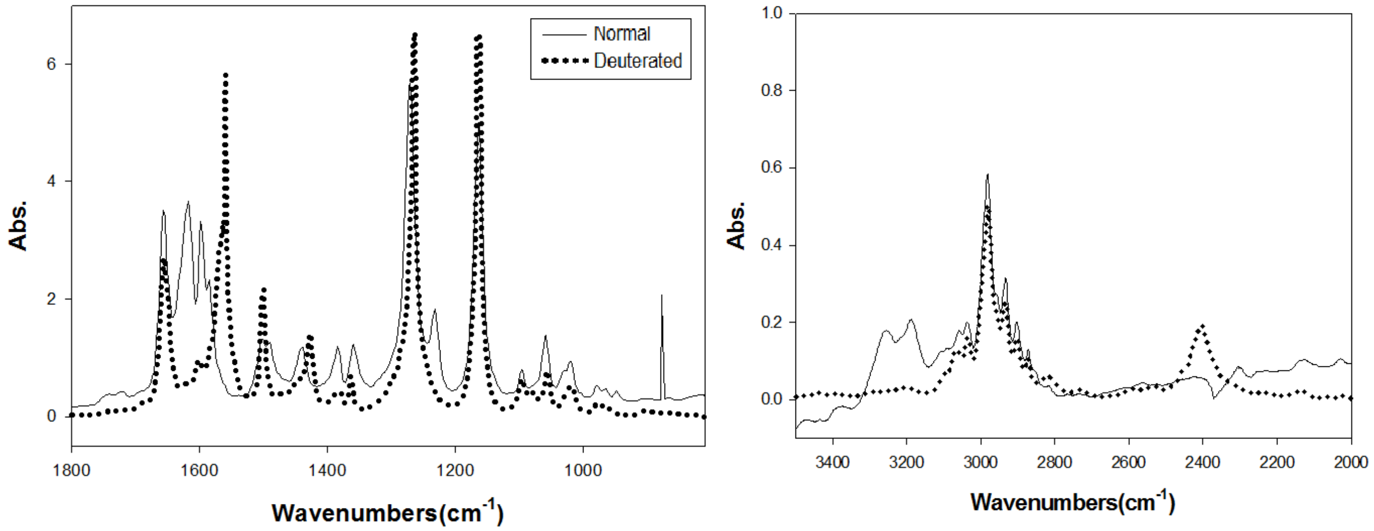

S7. IR spectrum of compound **16**.

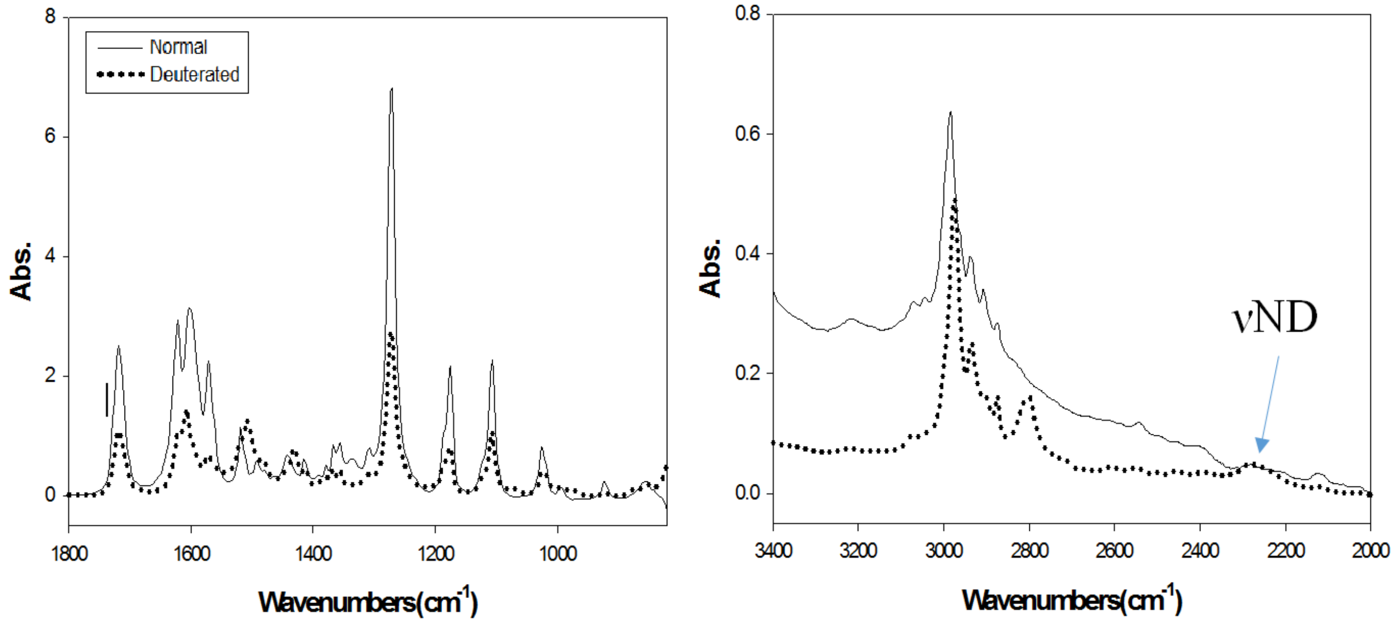

S8. IR spectrum of compound **44**.

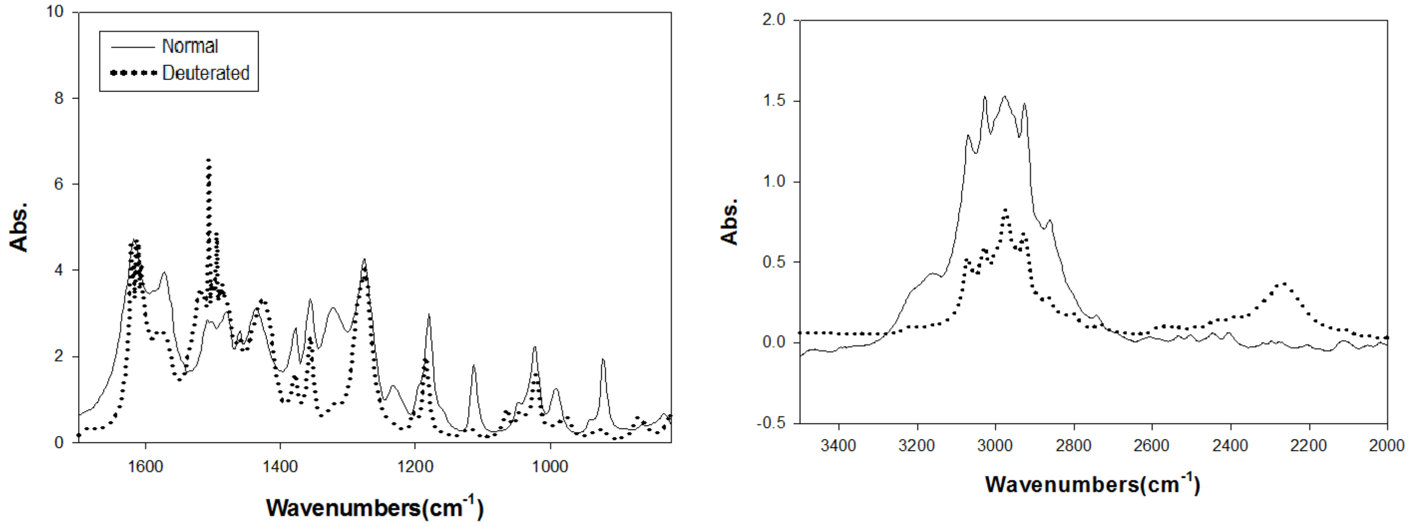

S9. IR spectrum of compound **8**.

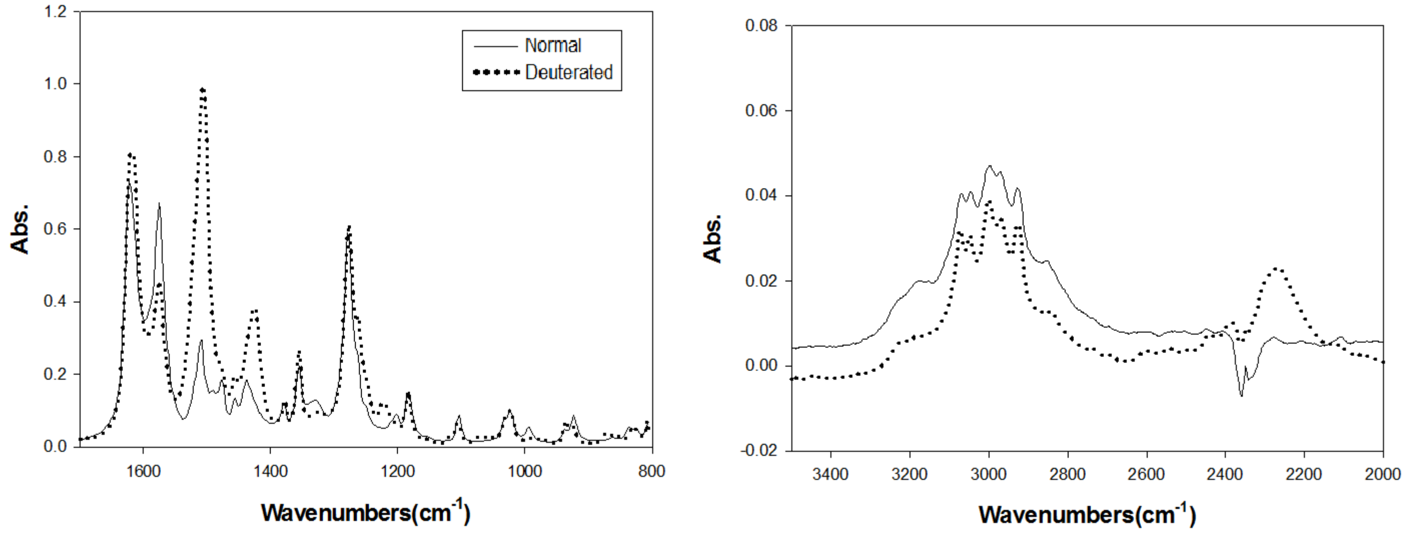

S10. IR spectrum of compound **9**.

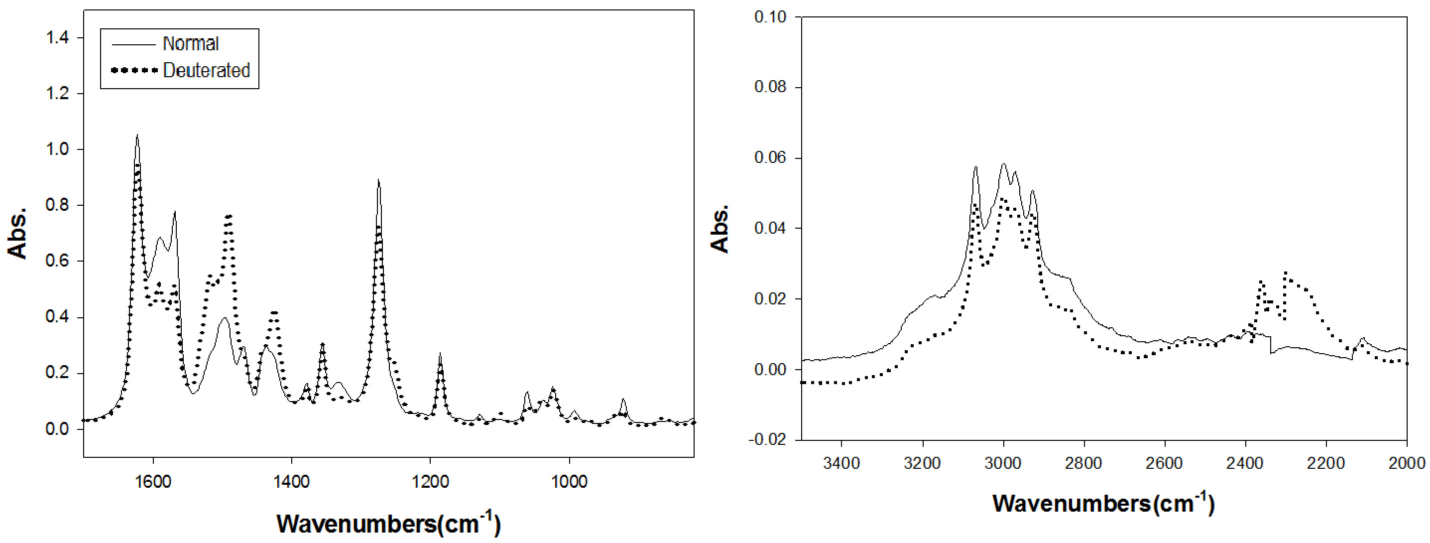

S11. IR spectrum of compound **10**.

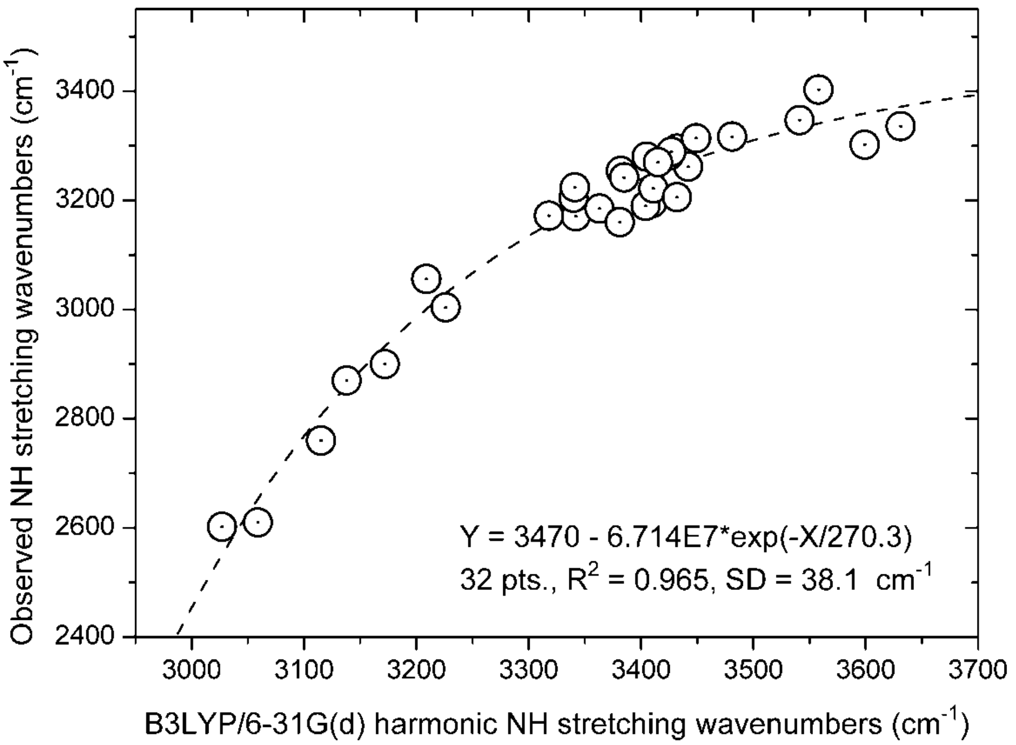

S12. Observed NH stretching wavenumbers (cm^{-1}) vs. harmonic values computed with B3LYP/6-31G(d). The points for compounds **25** and **26** are omitted and are not included in the correlation analysis.

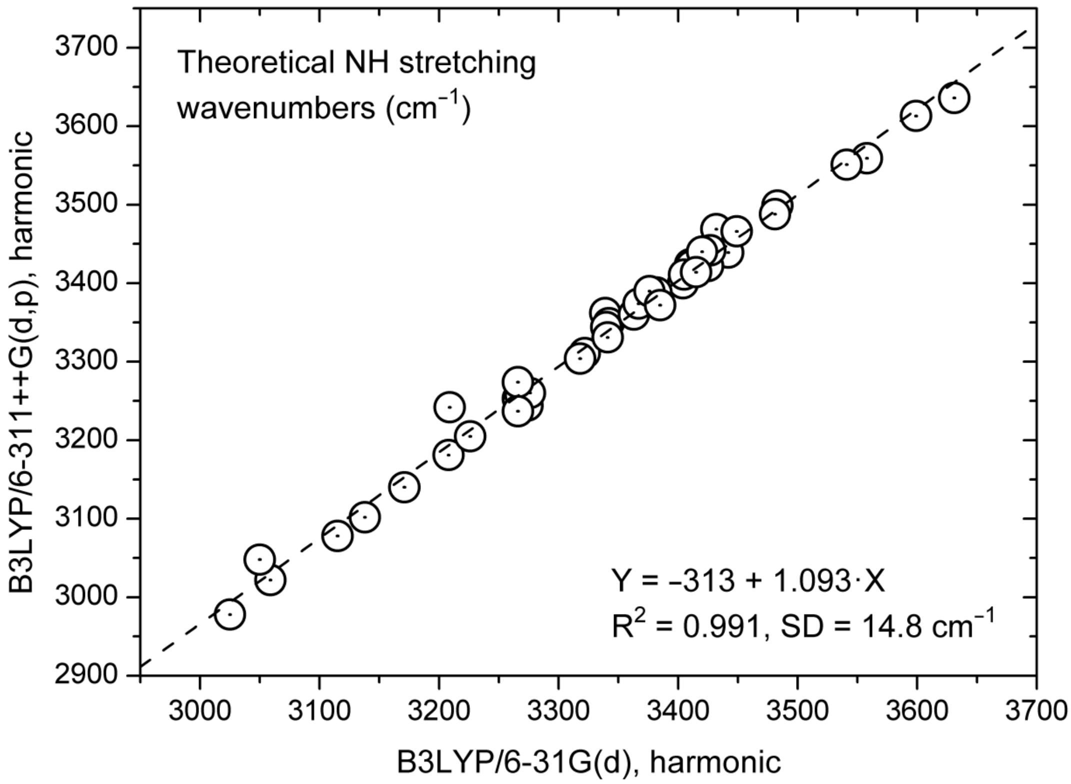

S13. Harmonic NH stretching wavenumbers (cm^{-1}) computed with B3LYP/6-311++G(d,p) against those obtained with B3LYP/6-31G(d).

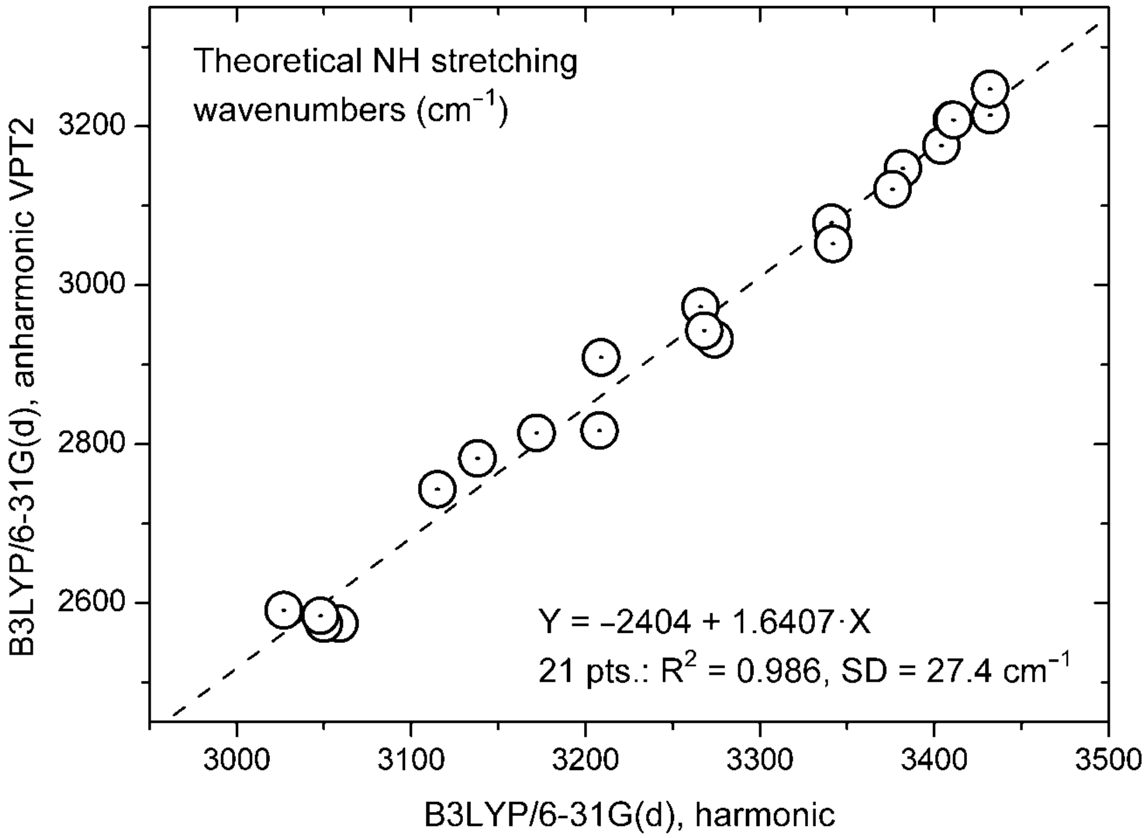

S14. B3LYP/6-31G(d) anharmonic VPT2 wavenumbers (cm^{-1}) vs. the corresponding harmonic ones.

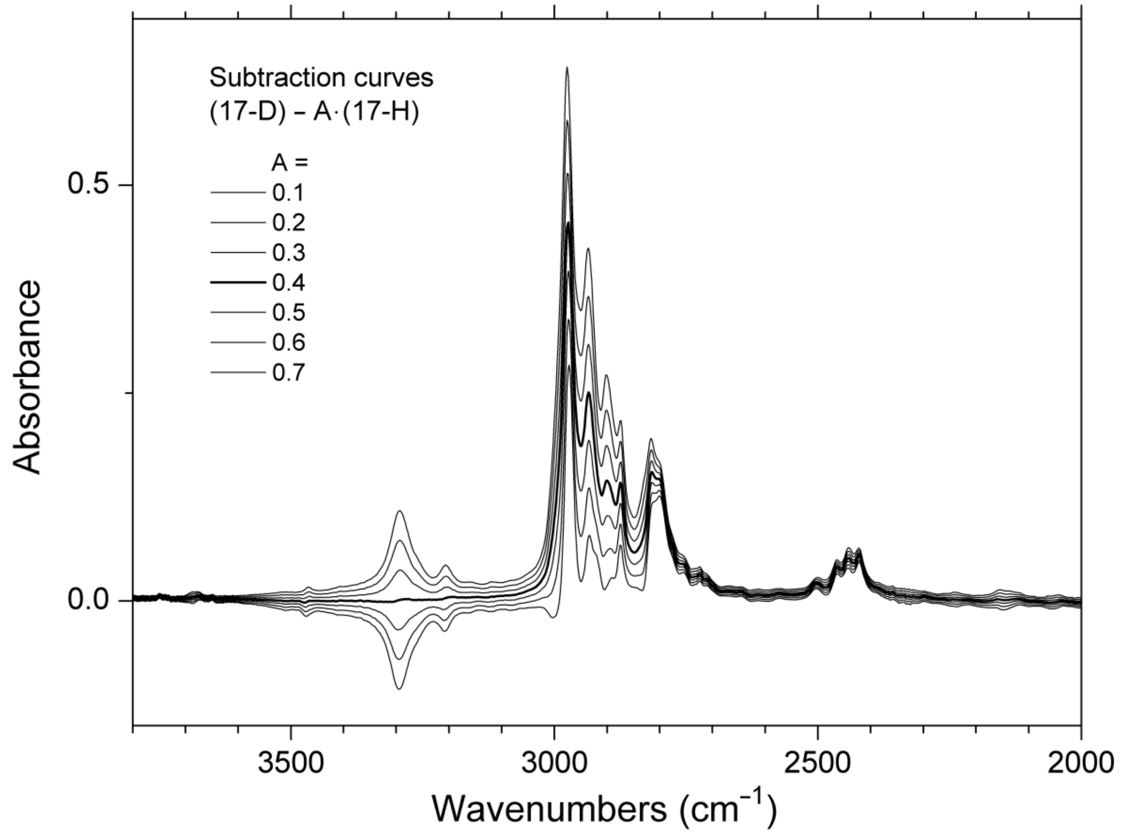

S15. Subtraction IR spectra of compound **17.**

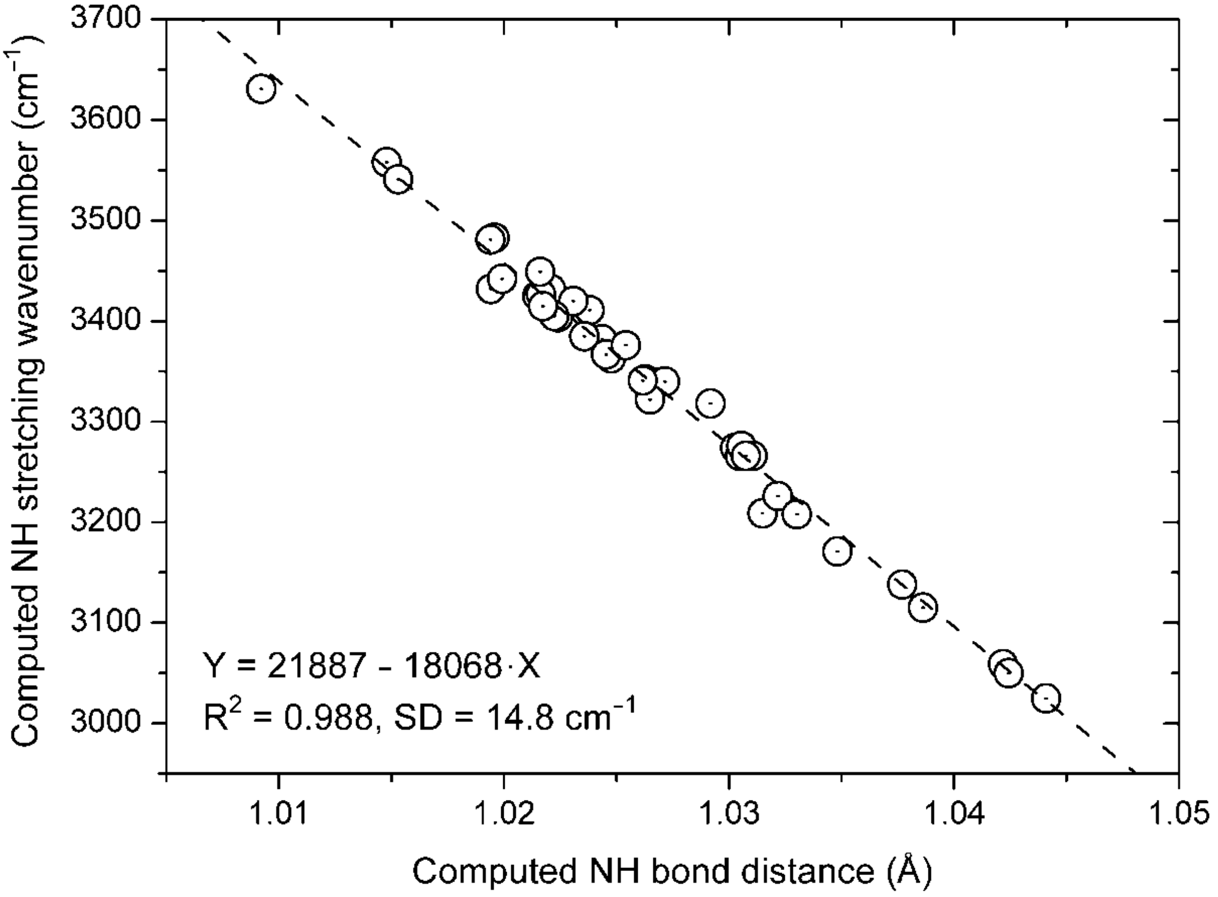

S16. Harmonic NH stretching wavenumbers vs. NH bond distances (B3LYP/6-31G(d)).

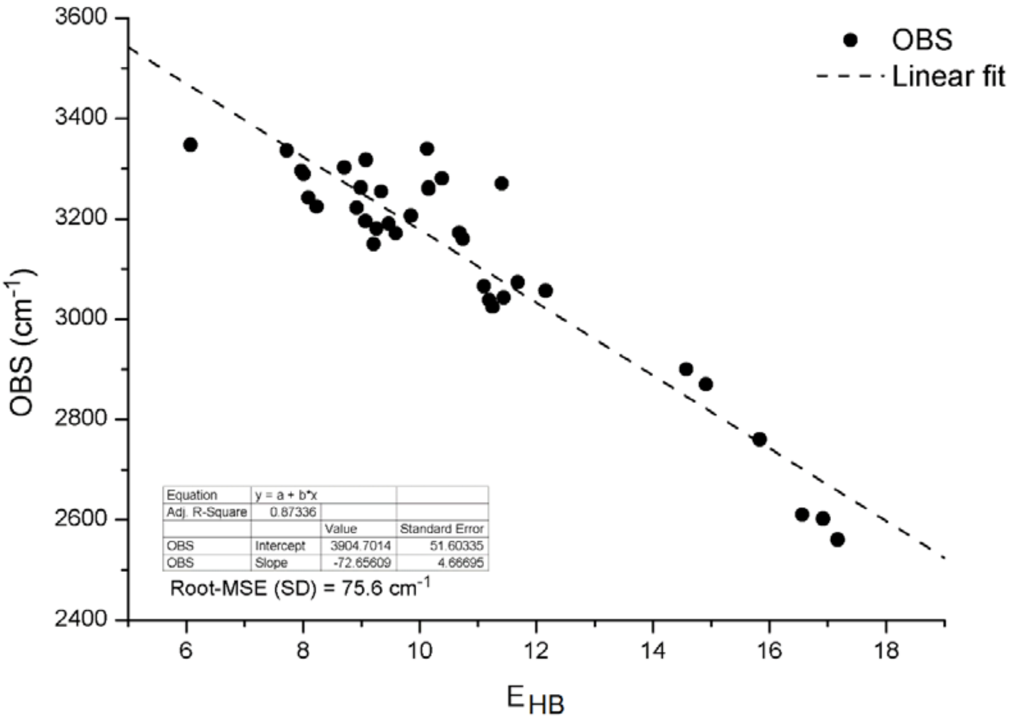

S17. Observed NH stretching wavenumbers vs. hydrogen bond energies, E_{HB} (kcal/mol), estimated according to E. Espinosa, E. Molins, C. Lecomte, Hydrogen bond strengths revealed by topological analyses of experimentally observed electron densities, Chem. Phys. Lett. 285 (1998) 170–173.

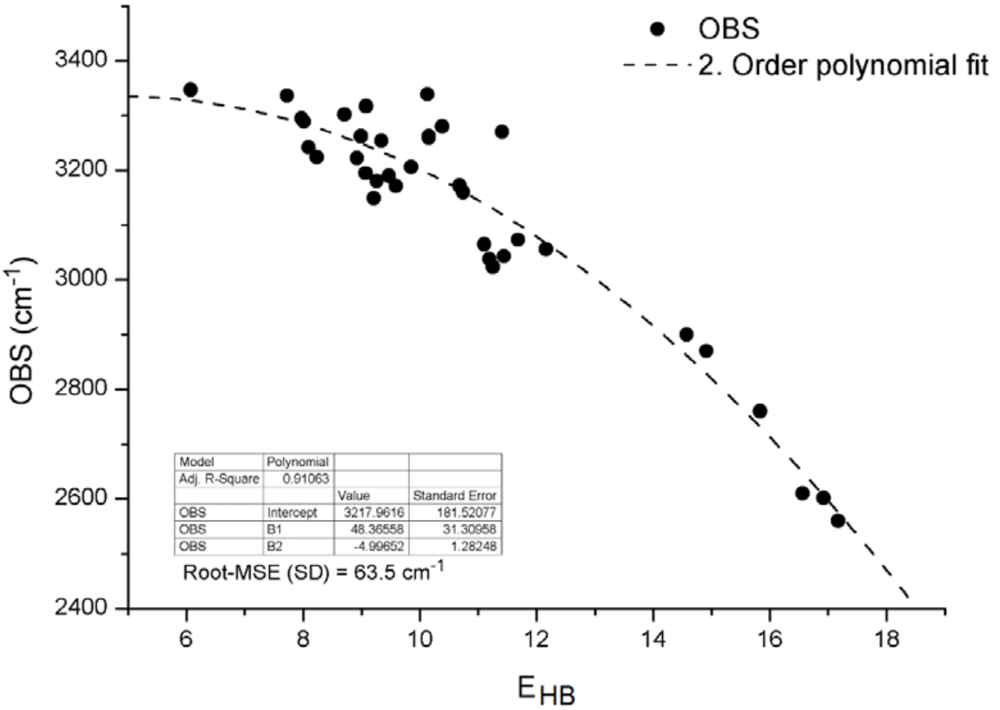

S18. Observed NH stretching wavenumbers vs. hydrogen bond energies, E_{HB} (kcal/mol), estimated according to E. Espinosa, E. Molins, C. Lecomte, Hydrogen bond strengths revealed by topological analyses of experimentally observed electron densities, Chem. Phys. Lett. 285 (1998) 170–173.

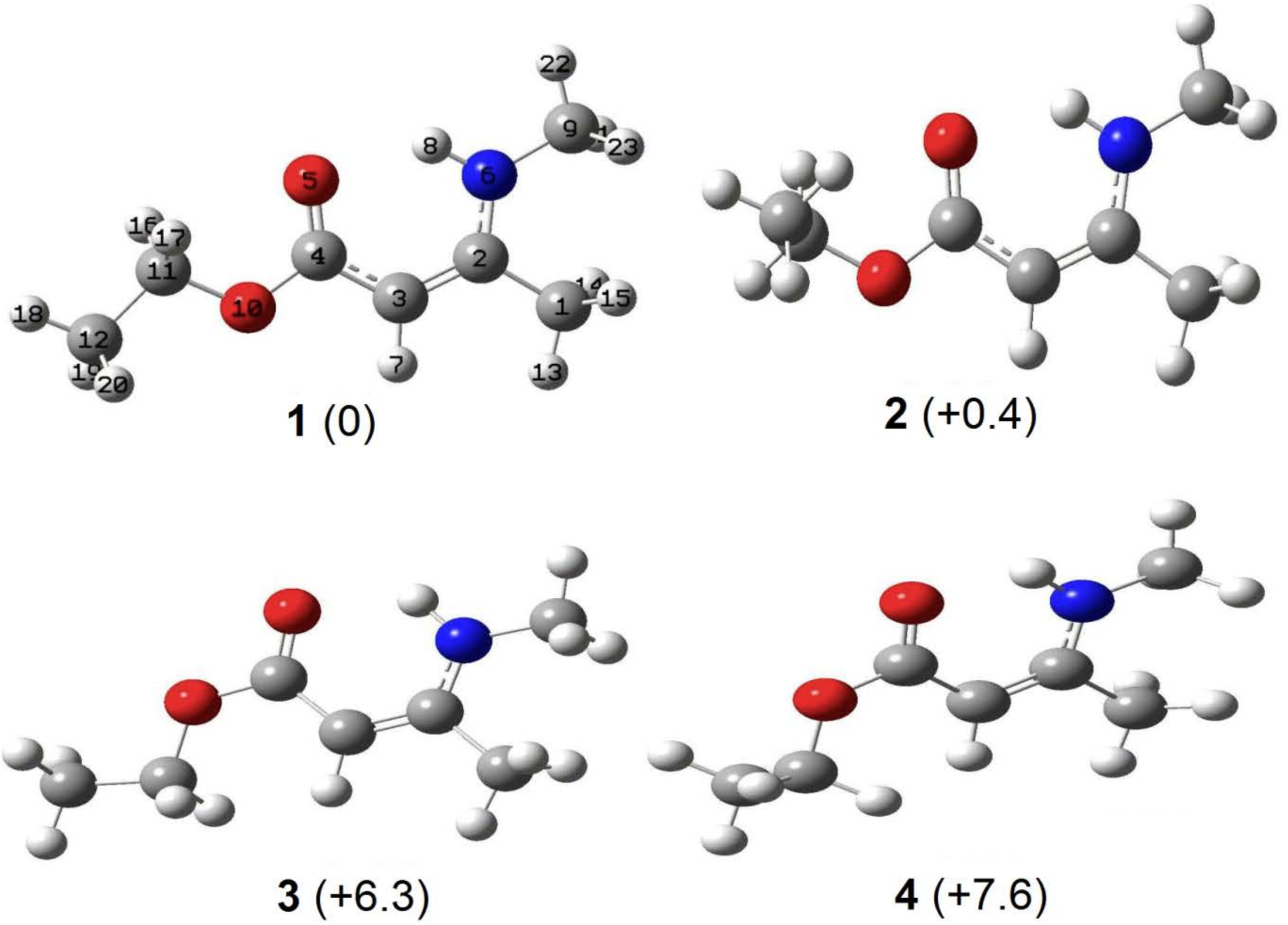

S19. Optimized molecular structures of (*Z*)-ethyl 3-(methylamino)but-2-enoate (**17**) computed with B3LYP/6-311++G(d,p) with relative energies in kcal/mol. The same harmonic NH stretching wavenumber is computed for all four structures, 3469 cm^{-1}. Two low-energy conformers, **1** and **2**, may coexist. This is consistent with previously reported results for β-keto esters by Tayyari et al. {1,2}.

{1} Tayyari, S.F.; Chahkandi, V.; Mehrani, S.; McClurg, R.W.; Keyes, C.A.; Sammelson, R.E. Conformational analysis, tautomerization, IR, Raman, and NMR studies of ethyl benzoylacetate. *J. Mol. Struct.* **2012**, *1015*, 74-85.

{2} Tayyari, S.F.; Naghavi, F.; Pojhan, S.; McClurg, R.W.; Sammelson, R.E. Conformational analysis, tautomerization, IR, Raman, and NMR studies of benzyl acetoacetate. *J. Mol. Struct.* **2011**, *987*, 241-254.

S20. Fundamental band assignments for **17** (17-H) and deuterated **17** (17-D).

17-H. Fundamental band assignments for **17**, conformations 1 and 2 (S19). **Freq.**, harmonic wavenumber in cm^{-1} (B3LYP/6-311++G(d,p)); **Int.**, corresponding IR intensity in km/mol; **Expt.**, wavenumber in cm^{-1} measured in CCl_4, relative intensity in parenthesis (s, strong; m, medium; w, weak; vw, very weak; sh, shoulder); **Assignment** (approximate): ν, stretching; δ, in-plane bending; ρ, in-plane rocking; π, out-of-plane bending; a, antisymmetric; s, symmetric; sci, scissor; Δ, in-plane chelated ring deformation; τ, torsion; for atom numbering, see S19, structure 1.

	**Conformation 1**		**Conformation 2**		**Expt.**	**Assignment**
	**Freq.**	**Int.**	**Freq.**	**Int.**		
					3465(w)	Summation band
1	3469	109	3469	110	3295(m)	νNH
2	3220	2	3222	2	3209(w)	νCH7
3	3136	11	3136	10	2983(m)	νa$C1H_3$
4	3121	17	3120	17	do.	νa$C9H_3$
5	3110	43	3124	30	2956(sh)	νa$C12H_3$, νaCH_2
6	3099	31	3104	12	do.	νa$C12H_3$, νsCH_2
7	3078	14	3077	13	2938(m)	νa$C1H_3$
8	3077	6	3092	30	do.	νaCH_2, νa$C12H_3$
9			3064	35	do.	νsCH_2
10	3048	32	3052	33	2899(m)	νa$C9H_3$
	3043	21			do.	νsCH_2
11	3033	23	3031	21	2870(sh)	νs$C12H_3$
12	3031	25	3030	26	do.	νs$C1H_3$
13	3006	80	3004	81	2826(w)	νs$C9H_3$
14	1685	331	1685	303	1650(s)	νaO=C-C=C, δCH7, νaN-C-C-C
15	1657	522	1657	530	1615(s)	δNH, νsO=C-C=C, νsN-C-C-C
16	1540	90	1539	102	1506(m)	νaN-C=C-C, δCH7, δNH, δa$C9H_3$, sciCH_2
17	1521	3				sciCH_2, δa$C12H_3$
			1509	21	1445(m)	δa$C9H_3$,sciCH_2, δa$C12H_3$
18	1508	17			do.	δa$C9H_3$, δCH7, δNH, νaN-C=C-C, sciCH_2
			1505	3	do.	δa$C12H_3$, δCH7, δNH, νaN-C=C-C, sciCH_2
19	1499	4	1490	16	1435(m)	δa$C12H_3$, sciCH_2
20	1498	19	1496	18	do.	δa$C1H_3$, δa$C9H_3$
21	1490	41	1487	17	do.	δa$C1H_3$, δNH, δa$C9H_3$
22	1485	7	1485	19	do.	δa$C12H_3$
23	1479	1	1478	1		δa$C1H_3$, δa$C9H_3$
24	1473	18	1470	20	do.	δs$C9H_3$, δNH, δa$C1H_3$
25	1424	0	1422	46	1380(m)	δa$C12H_3$, WCH_2, δs$C1H_3$
26	1421	53	1417	10	do.	δs$C1H_3$, δNH, δs$C12H_3$, WCH_2
27	1396	3	1398	5	do.	δs$C12H_3$, WCH_2, δNH, νsC=C
29	1391	75	1391	89	1360(m)	ρNH, δsCH_3, WCH_2, δCH7, νaC=C-N
29			1333	29	1301(sh)	WCH_2
30	1298	569	1298	469	1270(s)	δCH7, νaO10C4C3, νsN-C=C-C, νC2-CH_3, wCH_2
	1293	1				τCH_2, π$C12H_3$
31	1197	130	1196	159	1175(s)	δCH7, νN-CH_3, νaC-O-CH_2
32	1190	322	1194	21	do.	δCH7, νN-CH_3, ρ$C9H_3$, νaC-O-CH_2

33			1186	235	1165(m)	δCH7, νN-CH_3, ρC9H_3, νaC-O-CH_2
	1176	3				ρCH_2, ρC12H_3
34	1141	0	1139	5	1140(sh)	πC9H_3
35	1134	4			do.	ρC12H_3, δO-CH_2-CH_3
			1115	51	1097(w)	ρC12H_3
36	1105	45	1105	54	1083(m)	ρNH, νN-CH_3, ρC9H_3
37	1076	140	1068	97	1058(m)	νaOCH_2-C12H_3, νsC3-C4-O-CH_2, νC2-CH_3, ρC9H_3
38	1057	2	1057	3	1033(w)	πC1H_3
39	1029	6	1022	12	1011(w)	ρC1H_3, νaO-CH_2-C12H_3
40	993	21			977(w)	ρC1H_3, νsC-O-CH_2, δC2C3C4, νC-C12H_3
			974	19	967(w)	ρC1H_3, νsC-O-CH_2, δC2C3C4, νC-C12H_3
41	898	1	889	1		ρC12H_3, νsC-O-CH_2, δC2C3C4
42	838	6	836	5	825(w)	Δ, νN-C9H_3, νC-C1H_3, ρC12H_3

17-D. Fundamental band assignments for deuterated **17**. See heading to previous Table.

	**Conformation 1**		**Conformation 2**		**Expt.**	**Assignment**
	**Freq.**	**Int**	**Freq.**	**Int.**		
1	3137	11	3136	11	2982(m)	νaC1H_3
2	3121	16	3120	16	do.	νaC9H_3
3	3109	43	3124	30	2955(sh)	νaC12H_3, νaCH_2
4	3099	31	3104	12	do.	νaC12H_3, νsCH_2
5	3078	14	3077	13	2938(m)	νaC1H_3
6	3076	6	3092	30	do.	νaCH_2, νaC12H_3
7			3064	35	do.	νsCH_2
8	3048	32	3052	32	2900(sh)	νaC9H_3
	3043	21			do.	νsCH_2,
9	3033	23	3031	21	2870(sh)	νsC12H_3
10	3031	26	3030	26	do.	νsC1H_3
11	3007	77	3004	78	2815(w)	νsC9H_3
12	2545	71	2545	72	2431(w)	νND
13	2379	1	2380	1	2240(w)	νCD7
14	1679	316			1652(s)	νaO=C-C=C, νaN-C-C-C
			1679	291	do.	νaO=C-C=C, νsN-C-C-C
15	1597	601	1596	611	1560(s)	νsO=C-C=C, δND, νsN-C-C-C
16	1522	18	1519	28	1506 (vw)	δaC12H_3, δaC9H_3, sciCH_2
17	1518	11	1506	15	do.	sciCH_2, δaC9H_3, δaC12H_3
18	1499	4	1494	28	1440(sh)	δaC12H_3, sciC2H_2
19	1498	19	1496	18	do.	δaC1H_3, δaC9H_3
20	1491	17	1487	6	do.	δsC9H_3,νCN,sciCH_2
21	1485	7	1485	8	do.	δaC12H_3, sciCH_2
22	1480	103	1479	50	1435(m)	δaC1H_3
23	1478	1	1477	48	do.	δaC1H_3, δaC9H_3
24	1452	35	1450	32	1421(w)	δsC9H_3, δSC1H_3
25	1423	2	1418	9	1380(w)	δsC12H_3, WCH_2, δsC1H_3
26	1412	2	1411	3	1370(w)	δsC1H_3, δsC12H_3

27	1394	5	1396	14	1360(w)	δs$C12H_3$, WCH_2
28			1333	26	1326(w)	wCH_2
	1293	1			1302(vw)	wCH_2
29	1279	768	1278	638	1270(s)	νaO10C4C3,νsN-C=C-C, νC2-CH_3, wCH_2, ρND
30	1228	161	1228	138	1255(s)	ρND, νN-$C9H_3$, δC2C3C4, νaC-O-CH_2
31			1194	45	1175(sh)	ρCH_2
32	1179	54	1176	39	1165(m)	ρ$C9H_3$
	1176	3			do.	ρCH_2, ρ$C12H_3$
33	1137	0	1136	5	do.	νN-$C9H_3$
34	1134	4			do.	ρ$C12H_3$, δO-CH_2-CH_3
			1115	83	1095(m)	ρ$C12H_3$, δO-CH_2-CH_3
35	1095	206			1066(m)	ρ$C1H_3$, νaC-O-CH_2, δCD7
			1086	130	do.	ρ$C1H_3$, νaC-O-CH_2
36	1055	2	1055	2	1054(w)	π$C1H_3$
37	1037	21			1033(vw)	ρ$C1H_3$,νaO-CH_2-CH_3, δC2C3C4, νCCH_3
			1028	27	1010(vw)	ρ$C1H_3$,νaO-CH_2-CH_3, δC2C3C4, νCCH_3
38	991	34			998(sh)	ρ$C1H_3$, νsC-O-CH_2, δC2C3C4, νCCH_3
			974	31	975(w)	ρ$C1H_3$, νsC-O-CH_2, δC2C3C4, νCCH_3
39	961	5	962	6	964(w)	δND, νN-$C9H_3$
40	920	3	918	1	930(vw)	δCD7, δC-O-CH_2, δC2C3C4, ρ$C12H_3$

Notes:

The observed IR bands in non-deuterated **17** at 967(w), 1097(w), 1165(m), and 1301(sh) cm^{-1} may be due to the presence of conformation 2. The NH stretching of **17** is observed at 3295 cm^{-1}, on deuteration the band is observed at 2431 cm^{-1} (**S3, S15**). The corresponding NH band in 4-methylamino-3-penten-2-one, APO-NMe, was observed at 3171 cm^{-1} by H. Raissi et al. {3}. This value indicates that the IHB strength of **17** is weaker than that of APO-NMe. Hence, replacement of the β-methyl group in APO-NMe with an OEt group (to yield **17**) decreases the IHB strength. - The spectrum of **17** in CCl_4 shows a weak band at 3465 cm^{-1}, this may be a summation band. In 4-amino-3-penten-2-one, APO, a corresponding band is seen at 3240 cm^{-1}{4}.

{3} Raissi, H.; Moshfeghi, E.; Farzad, F.; Vibrational assignment, structure and intramolecular hydrogen bond of 4-methylamino-3-penten-2-one. *Spectrochim. Acta A* **2005**, *62*, 1004-1015.

{4} Tayyari, S.F.; Raissi, H.; Tayyari, F. Vibrational assignment of 4-amino-3-penten-2-one. *Spectrochim. Acta A* **2002**, *58*, 1681-1695.

S21. Materials section: Synthesis, characterization, and NMR spectra.

All chemicals, unless stated otherwise, were purchased from Sigma-Aldrich, TCI, Chemicals and Alfa Aesar and were used without further purification. Solvents used were HPLC grade and dried on molecular sieves (4 Å) at least 2 days before use. Analytical thin-layer chromatography was done on Merck Alufolien SiO_2 60 F254 0.2 mm thick precoated TLC plates. Column chromatography was performed using SiO_2 from VWR 40−63 µm. ^{1}H and ^{13}C NMR spectra were recorded on a Bruker 500 MHz Ultrashield Plus 500 spectrometer. ^{1}H, ^{13}C, and ^{19}F NMR spectra were also recorded at 500, 125, and 470 MHz, respectively, on a Bruker Avance 3 spectrometer with a BBFO probe. All chemical shifts (δ) are quoted in ppm, and all coupling constants (J) are in Hertz (Hz). The following abbreviations are used for multiplicity for NMR resonances: s = singlet, bs = broad singlet, d = doublet, t = triplet, q = quartet and m = multiplet. HRMS LC/MS were recorded on a Dionex Acclaim RSLC 120 C18 2.2 µm 120 Å 2.150 mm column maintained at 40 ^{o}C carried out on a Bruker MicrOTOF-QII-system with ESI system.

Compounds **6**, **14**, **16**, **17**, **19**, and **45** were prepared by a modified procedure based on Martin, D.F.; Janusonis, G.A.; Martin, B.B. Stabilities of Bivalent Metal Complexes of some B-Ketoimines, *J. Am. Chem. Soc.* **1961**, *83*, 73-75.

Modified procedure: To a stirred solution of 1,3-dicarbonyl compound (5.0 mmol of 2,4-pentanedione, 3-methyl-2,4-pentanedione, ethyl 2-acetoacetate or ethyl 2-methylacetoacetate) and amine (5.0 mmol of methylamine or anilines) in absolute ethanol (15 mL) was added glacial acetic acid (0.01 mL) at room temperature. The reaction mixture was stirred at 80 ^{o}C and the progress of the reaction was monitored by TLC. After complete conversion of the starting material, the solvent was removed under reduced pressure and the crude product was purified by column chromatography using silica gel using a gradient of organic solvents as eluent. Evaporation of solvent under reduced pressure affords the pure compound.

The synthesis of **8**, **9**, **10**, and **11** has been described in Zheglova, D.Kh.; Genov, D.G.; Bolvig, S.; Hansen, P.E. Deuterium Isotope Effects on ^{13}C Chemical Shifts of Enaminones. *Acta Chem. Scand.* **1997**, *51*, 1016-1023. Additional ^{13}C NMR data and high resolution MS data are given below .

(Z)-4-(phenylamino)pent-3-en-2-one **6**:
The compound was isolated as a pale yellow solid after flash column chromatography on silica using dichloromethane/ethyl acetate (20:1) as eluent. In 94% yield. ^{1}H NMR (500 MHz, $CDCl_3$): δ 12.45 (brs, 1H, NH),7.34-7.31 (m, 2H), 7.19-7.16 (m, 1H), 7.10-7.09 (m, 2H), 5.18 (s, 1H), 2.09 (s, 3H), 1.98 (s, 3H). ^{13}C NMR (125 MHz, $CDCl_3$): δ 196.23, 160.36, 138.83, 129.18, 125.65, 124.84, 97.70, 29.26, 19.93. NMR results are similar to those of Ref. [*i*]. HRMS-ESI (m/z): $[M + H]^+$ calcd. for $C_{11}H_{14}NO$: 176.0997, found: 176.0949.

(Z)-4-(o-tolylamino)pent-3-en-2-one **8**:
^{1}H NMR (500 MHz, $CDCl_3$): δ 12.33 (brs, 1H, NH), 7.23-7.21 (m, 1H), 7.17-7.13 (m, 2H), 7.08-7.06 (m, 1H), 5.19 (s, 1H), 2.28 (s, 3H), 2.10 (s, 3H), 1.86 (s, 3H). ^{13}C NMR (125 MHz, $CDCl_3$): δ 196.12, 161.26, 137.58, 133.80, 130.87, 130.87, 126.48, 126.48, 97.12, 29.16, 19.70, 18.15. The NMR results are similar to those of Ref. [*ii*]. HRMS-ESI (m/z): $[M + H]^+$ calcd. for $C_{11}H_{14}NO$: 176.0997, found: 176.0949.

(Z)-4-(2-fluorophenylamino)pent-3-en-2-one **9:**
^{1}H NMR (500 MHz, $CDCl_3$): δ 12.23 (brs, 1H, NH), 7.17 (m, 2H), 7.12 (m, 2H), 5.26 (s, 1H), 2.11 (s, 3H), 1.94 (s, 3H). ^{13}C NMR (125 MHz, $CDCl_3$): δ 196.88, 160.61, 156.80 (J=247.5), 127.26 (J=7.61), 127.14, 127.04 (J=12.45) , 124.44 (J=4.34), 116.39 (J=20.19), 98.26, 29.34, 19.60. NMR results are similar to those of Refs. [*i*] and [46]. ^{19}F NMR (470 MHz, $CDCl_3$): δ 123.65 ppm (Reference hexafluorobenzene). HRMS-ESI (m/z): $[M + H]^+$ calcd. for $C_{11}H_{13}FNO$: 194.0903, found: 194.0988.

(Z)-4-(2-chlorophenylamino)pent-3-en-2-one **10:**
^{1}H NMR (500 MHz, $CDCl_3$): δ 12.42 (brs, 1H, NH), 7.46-7.44 (m, 1H), 7.25-7.20 (m, 2H), 7.17-7.14 (m, 1H), 5.27 (s, 1H), 2.13 (s, 3H), 1.95 (s, 3H). ^{13}C NMR (125 MHz, $CDCl_3$): δ 196.87, 159.84, 136.46, 130.22, 129.98, 127.25, 126.92, 126.90, 98.52, 29.35, 19.88. HRMS-ESI (m/z): $[M + H]^+$ calcd. for $C_{12}H_{16}ClNO$: 225.0920, found, 225.0952. The ^{13}C data are different from those of Ref. [*iii*]. The spectrum contains a small degradation product, which is 2-chloroaniline. This proves that the compound is the correct one.

(Z)-4-(2,6-dimethylphenylamino)pent-3-en-2-one **11:**
^{1}H NMR (500 MHz, $CDCl_3$): δ 11.92 (brs, 1H, NH), 7.08 (m, 3H), 5.20 (s, 1H), 2.19 (s, 3H), 2.10 (s,3H), 1.62 (s, 3H). ^{13}C NMR (125 MHz, $CDCl_3$): δ 195.62, 162.95, 136.64, 136.24, 135.65, 128.30, 127.51, 95.88, 29.14, 18.34. NMR results are similar to those of Ref. [*i*]. HRMS-ESI (m/z): $[M + H]^+$ calcd. for $C_{13}H_{18}NO$: 204.1310, found: 204.1394.

(Z)-3-methyl-4-(methylamino)pent-3-en-2-one **14:**
The compound was isolated as a light amber oil after flash column chromatography on silica using *n*-hexane/ethyl acetate (1:1) as eluent. In 74% yield. ^{1}H NMR (500 MHz, $CDCl_3$): δ 11.80 (brs, 1H, NH), 2.88 (d, J=5.2 Hz, 3H), 2.06 (s, 3H), 1.91 (s, 3H), 1.78 (s, 3H). The ^{1}H NMR results are similar to those of Ref. [*iv*]. ^{13}C NMR (125 MHz, $CDCl_3$): δ 194.35, 163.15, 97.97, 29.84, 28.31, 14.93, 14.58. HRMS-ESI (m/z): $[M + H]^+$ calcd. for $C_{13}H_{18}NO$: 204.1310, found: 204.1394.

(Z)-ethyl 3-(phenylamino)but-2-enoate **16:**
The compound was isolated as creamy solid after flash column chromatography on silica using dichloromethane/ethyl acetate (20:1) as eluent. In 70% yield. ^{1}H NMR (500 MHz, $CDCl_3$): δ 10.39 (brs, 1H, NH), 7.31 (m, 2H), 7.14 (m, 1H), 7.08 (m,2H), 4.69 (s, 1H) 4.15 (q, 2H), 1.99 (s, 3H), 1.28 (t, 3H). ^{13}C NMR (125 MHz, $CDCl_3$): δ 170.44, 158.96, 139.42, 129.58, 124.96, 124.45, 86.13, 58.80, 20.36, 14.66. The NMR results are similar to those of Ref. [*v*]. HRMS-ESI (m/z): $[M + H]^+$ calcd. for $C_{12}H_{16}NO_2$: 206.1181, found: 206.1172.

(Z)-ethyl 3-(methylamino)but-2-enoate **17:**
The compound was isolated as a light amber oil after flash column chromatography on silica using n-hexane/ethyl acetate (1:1) as eluent. In 61% yield. ^{1}H NMR (500 MHz, $CDCl_3$): δ 8.46 (brs, 1H, NH), 4.45 (s, 1H), 4.07 (q, 2H), 2.89 (s, 3H), 1.90 (s, 3H), 1.23 (t, 3H). ^{13}C NMR (125 MHz, $CDCl_3$): δ 170.79, 162.91, 81.99, 58.38, 29.67, 19.28, 14.76. The NMR results are similar to those of Ref. [*vi*]. HRMS-ESI (m/z): $[M + H]^+$ calcd. for $C_7H_{14}NO_2$: 144.2024.1310, found: 144.1037.

(Z)-ethyl 2-methyl-3-(methylamino)but-2-enoate **19:**
The compound was isolated as a colorless oil after flash column chromatography on silica using *n*-hexane/ethyl acetate (1:1) as eluent. In 68% yield. ^{1}H NMR (500 MHz, $CDCl_3$): δ 9.14 (brs, 1H, NH), 4.08 (q, J=7.1, 2H), 2.88 (d, J=5.2, 3H), 1.94 (s, 3H), 1.77 (s, 3H), 1.25 (t, J=7.1, 3H). ^{13}C NMR (125 MHz, $CDCl_3$): δ 170.96, 160.39, 86.18, 58.48, 29.85, 14.83, 14.55, 12.51. HRMS-ESI (m/z): $[M + H]^+$ calcd. for $C_8H_{16}NO_2$: 158.1181, found: 158.1162.

(Z)-3-methyl-4-(phenylamino)pent-3-en-2-one **45:**
The compound was isolated as off white solid after flash column chromatography on silica using *n*-hexane/ethyl acetate (1:1) as eluent. In 78% yield. ^{1}H NMR (500 MHz, $CDCl_3$): δ 13.44 (brs, 1H, NH),7.28 (t, 2H), 7.12 (t, 1H), 7.02 (d, J=7.4, 2H), 2.18 (s, 3H), 1.98, (s, 3H), 1.91 (s, 3H). ^{13}C NMR (125 MHz, $CDCl_3$): δ 196.71, 158.50, 139.63, 129.66, 125.27, 101.15, 28.87, 17.21, 15.02. The NMR results are similar to those of Ref. [*vii*]. HRMS-ESI (m/z): $[M + H]^+$ calcd. for $C_{13}H_{18}NO_2$: 220.1337, found: 220.1348.

i. Schilling, W.; Zhang, Y.; Sahoo, P.K.; Sarkar, S.K.; Gandhi, S.; Roesky, H.W.; Das, S. Nature inspired singlet oxygen generation to access α-amino carbonyl compounds via 1,2-acyl migration. *Green Chemistry*, **2021**, *23*, 379-387.

ii. Prabakaran, K.; Sivakumar, M.; Perumal, M.S. A Simple, Efficient Green Protocol for the Synthesis of β-Enaminone and Enamino Ester Derivatives by Using Onion Extract as Green Catalyst. *ChemistrySelect*, **2017**, *2*, 2363-2372.

iii. Olejník, R.; Padělková, Z.; Horáček, M.; Růžička, A. Structure of *β*-diketiminates and *β*-aminoketones made from anisidines or chloroanilines: tin and lithium complexes. *Main Group Metal Chemistry* **2012**, *35*,13-27.

iv. Mikysek, T.; Kvapilová, H.; Doušová, H.; Josefík, F.; Šimůnek, P.; Růžičková, Z.; Ludvík, J. Synthesis, electrochemical, structural and theoretical study of new derivatives of OABAN and OABAO heterocycles *Inorg. Chim. Acta* **2017**, *455*, 465-472.

v. Qiao, W-Z.; Song, T-Q.; Cheng, P.; Zhao, B. Highly Selective Enamination of β-ketoesters Catalyzed by Interlocked [Cu8] and [Cu18] Nanocages. *Angew. Chem., Intern. Ed.*, **2019**, *58*, 13302-13307.

vi. Manzi, J.A.; Knapp, C.E; Carmalt, C.J. Aerosol-Assisted Chemical-Vapour Deposition of Zinc Oxide from Single-Source β-Iminoesterate Precursors. *Eur. J. Inorg. Chem.*, **2015,** *22*, 3658-3665.

vii. Chen, X.; She, J.; Shang, Z.; Wu, J.; Wu, H.; Zhang, P. Synthesis of pyrazoles, diazepines, enaminones, and enamino esters using 12-tungstophosphoric acid as a reusable catalyst in water. *Synthesis*, **2008**, *21*, 3478-3486.

NMR:

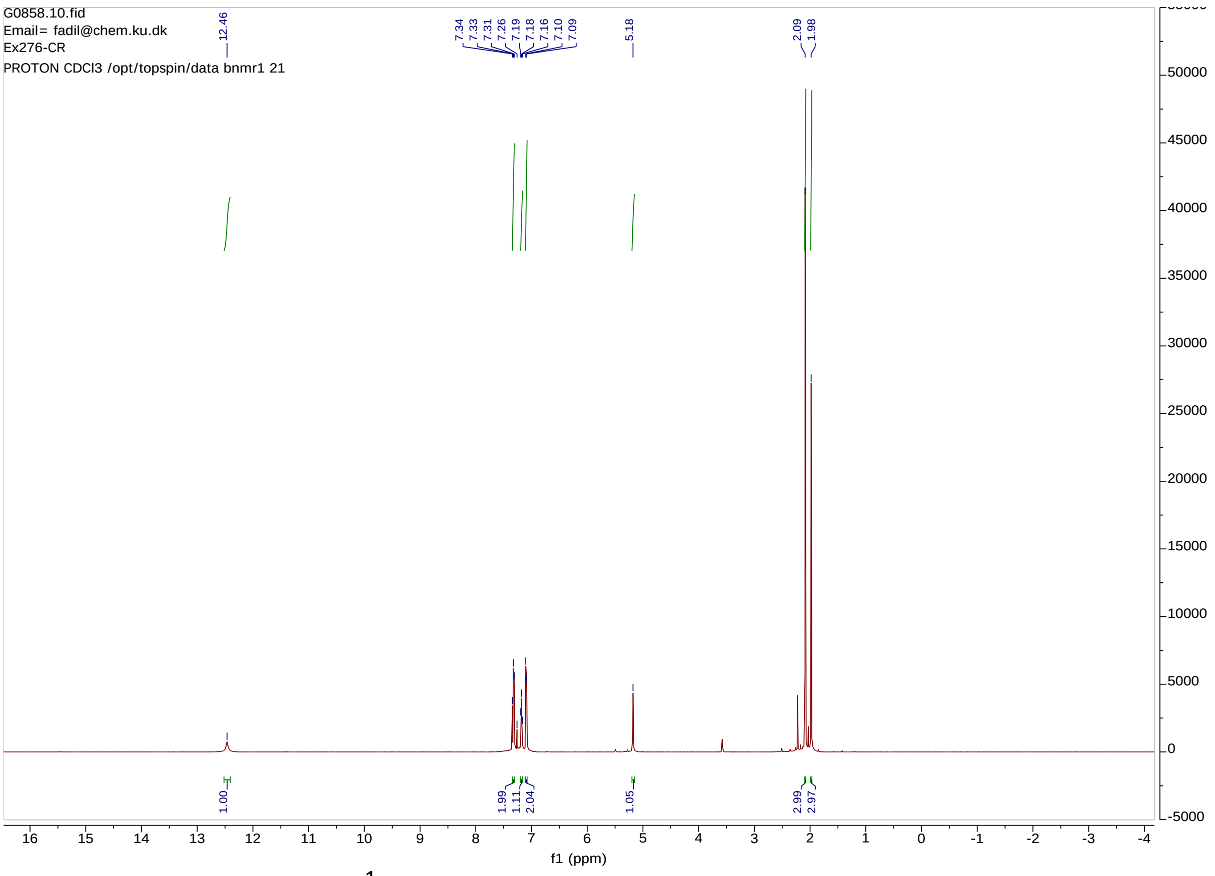

^{1}H NMR spectrum of compound **6**

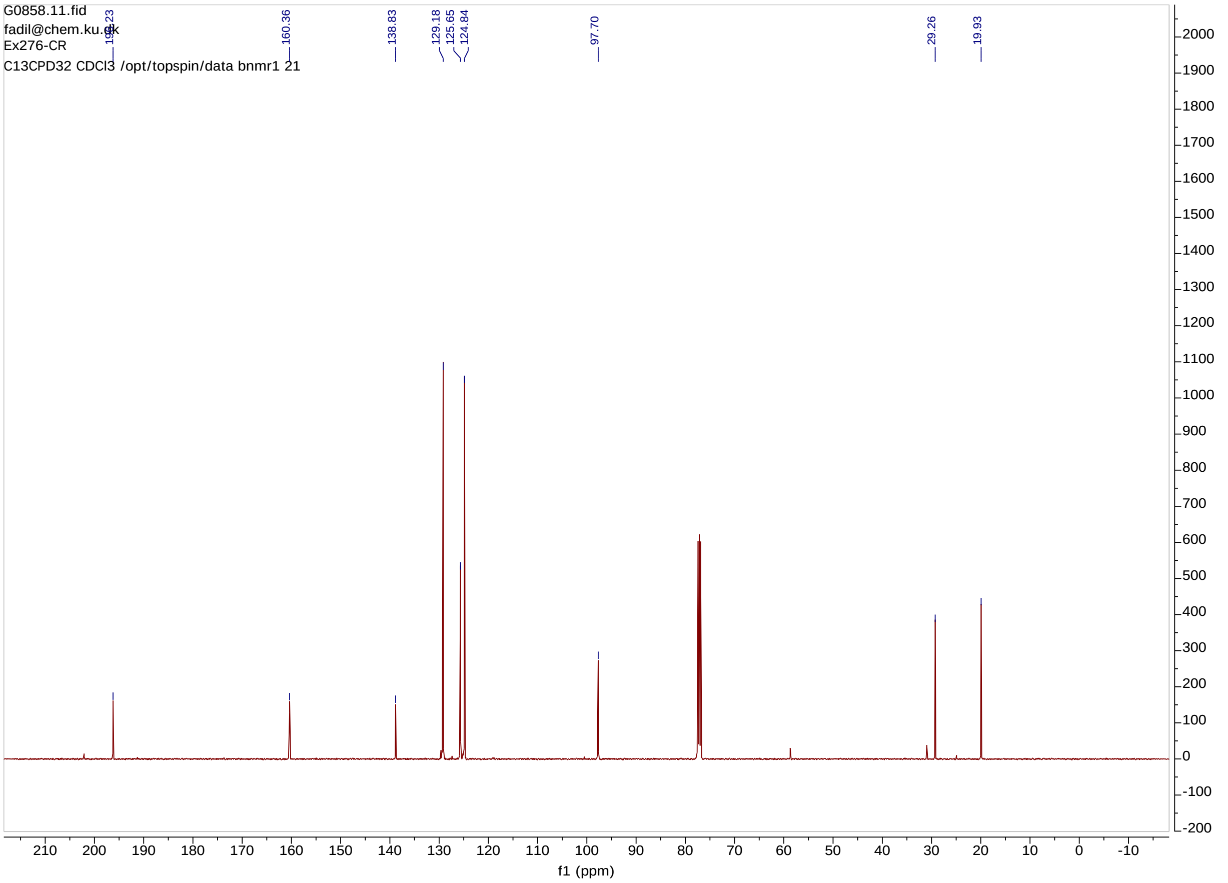

^{13}C NMR spectrum of compound **6**

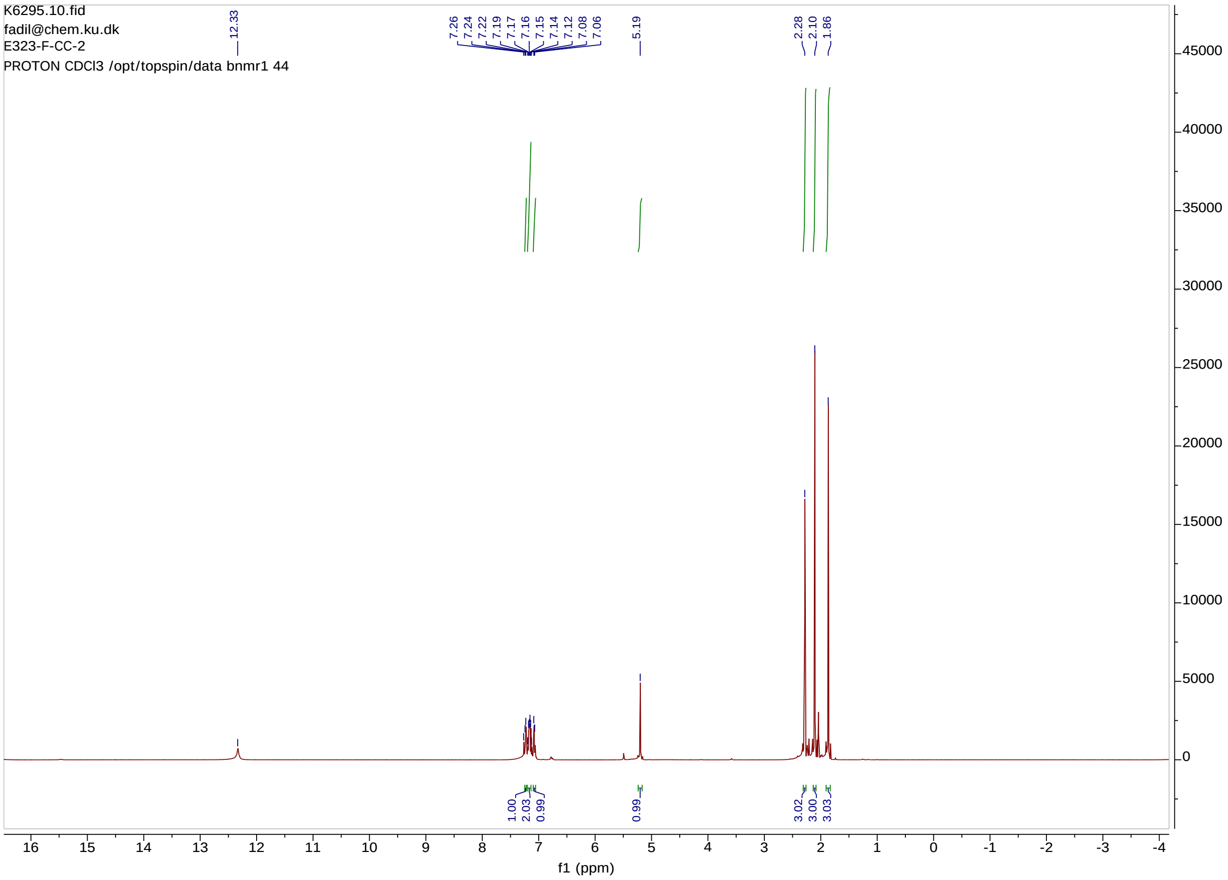

^{1}H NMR spectrum of compound **8**

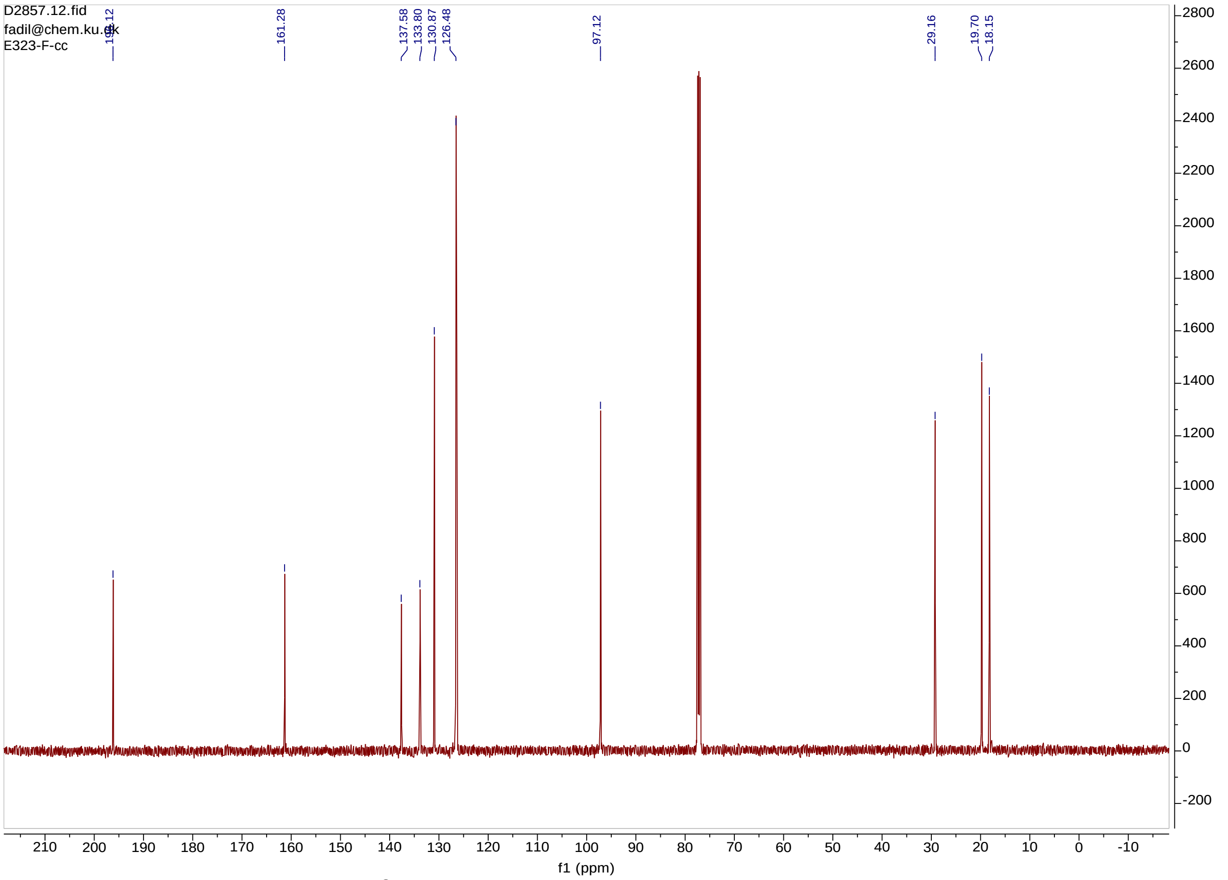

^{13}C NMR spectrum of compound **8**

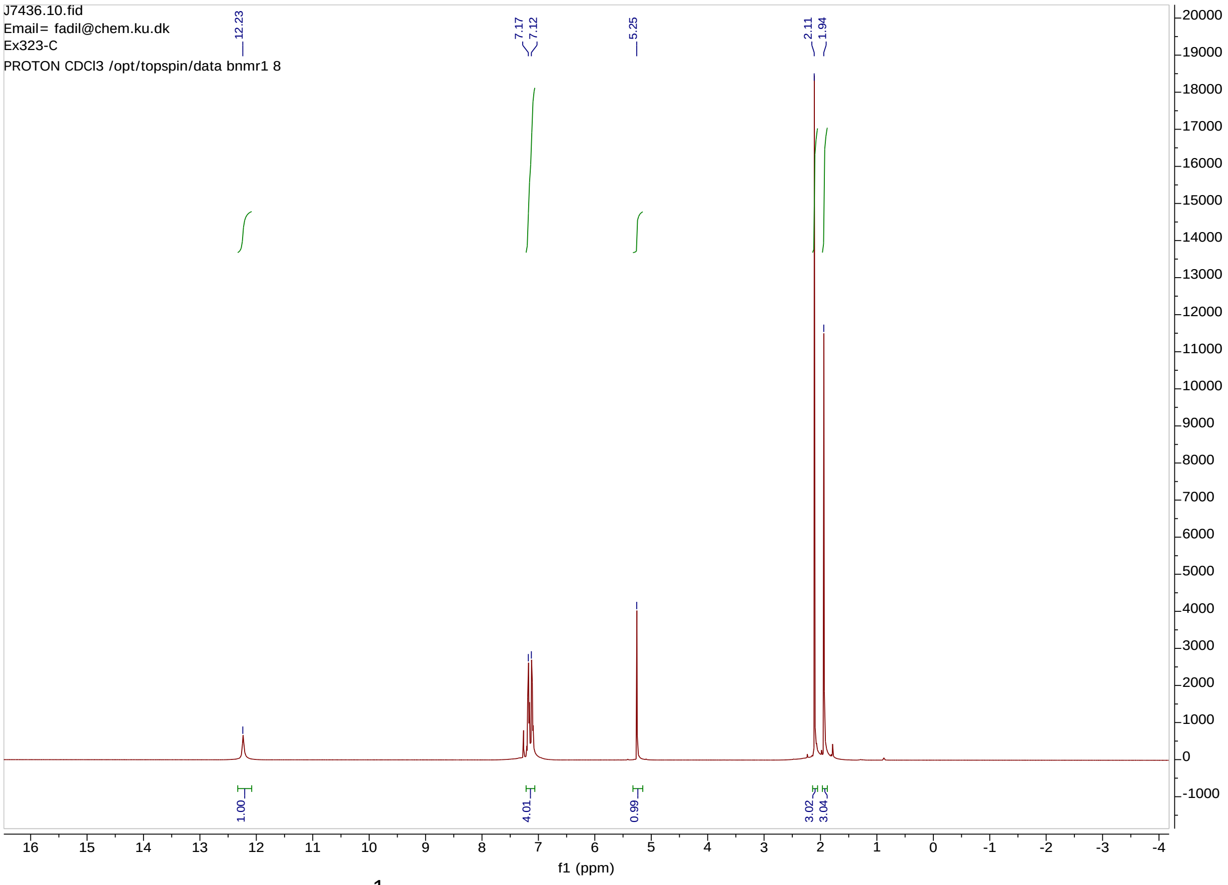

^{1}H NMR spectrum of compound **9**

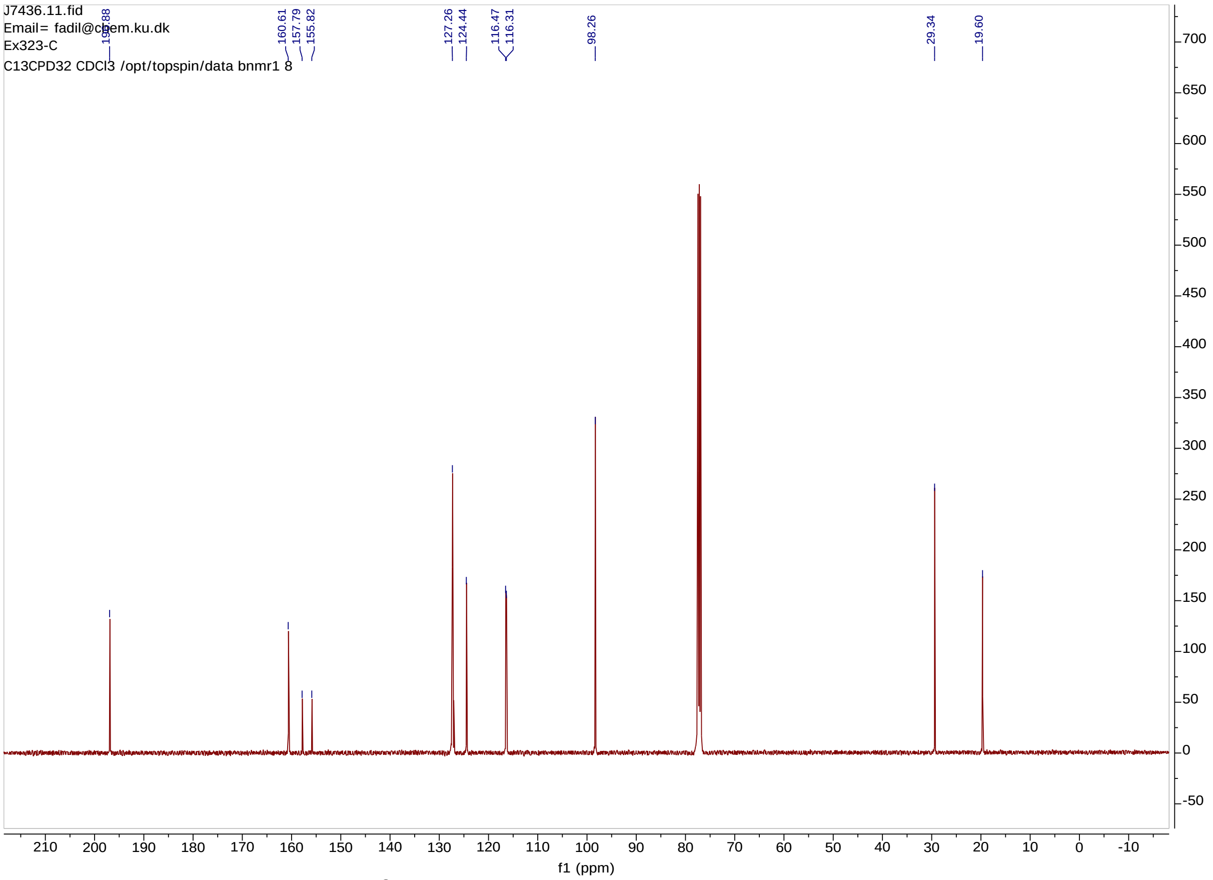

^{13}C NMR spectrum of compound **9**

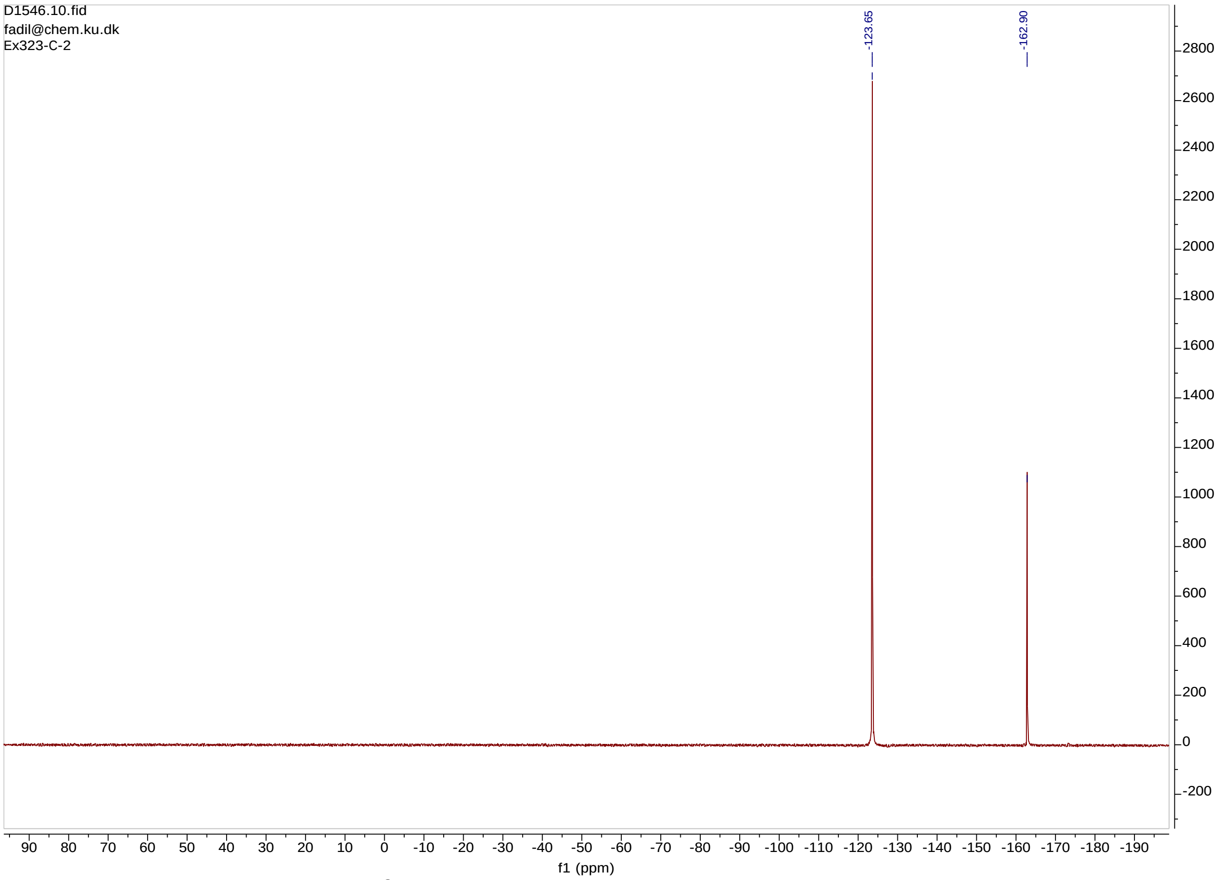

^{19}F NMR spectrum of compound **9**

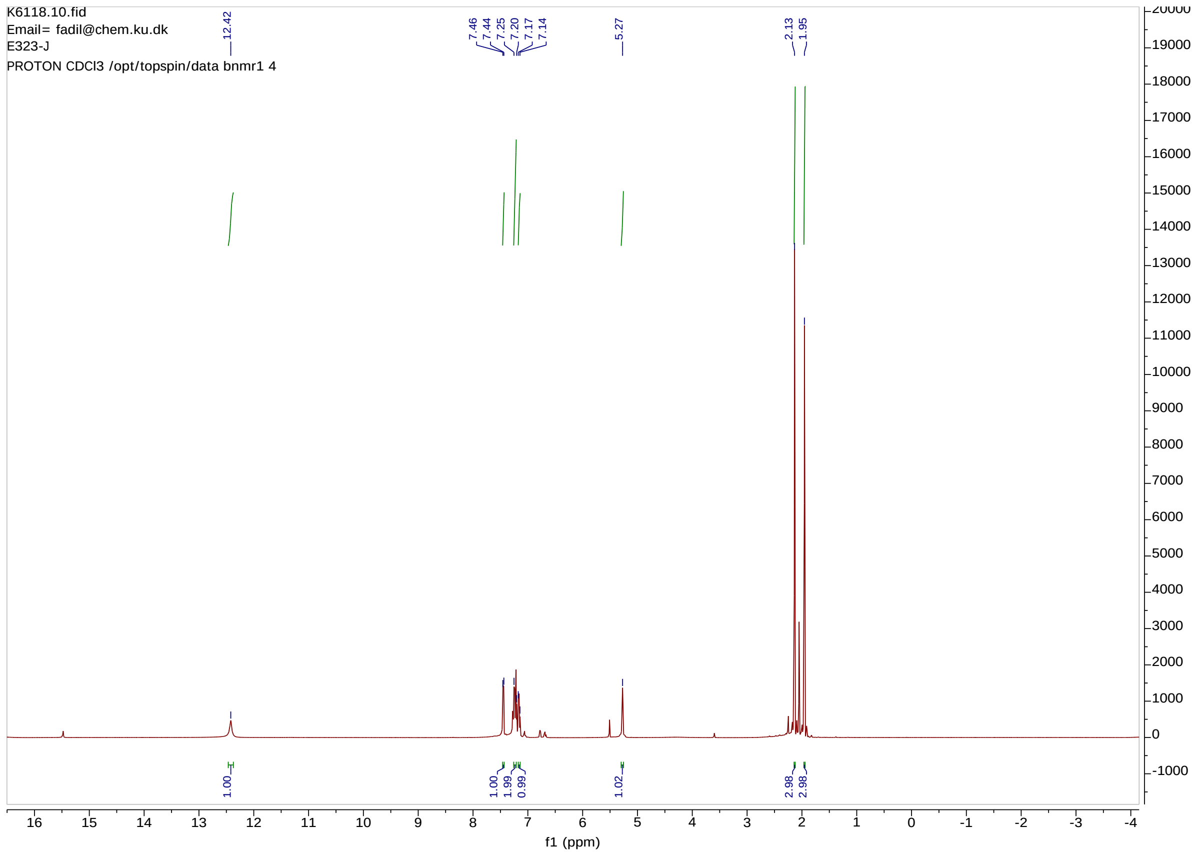

^{1}H NMR spectrum of compound **10**

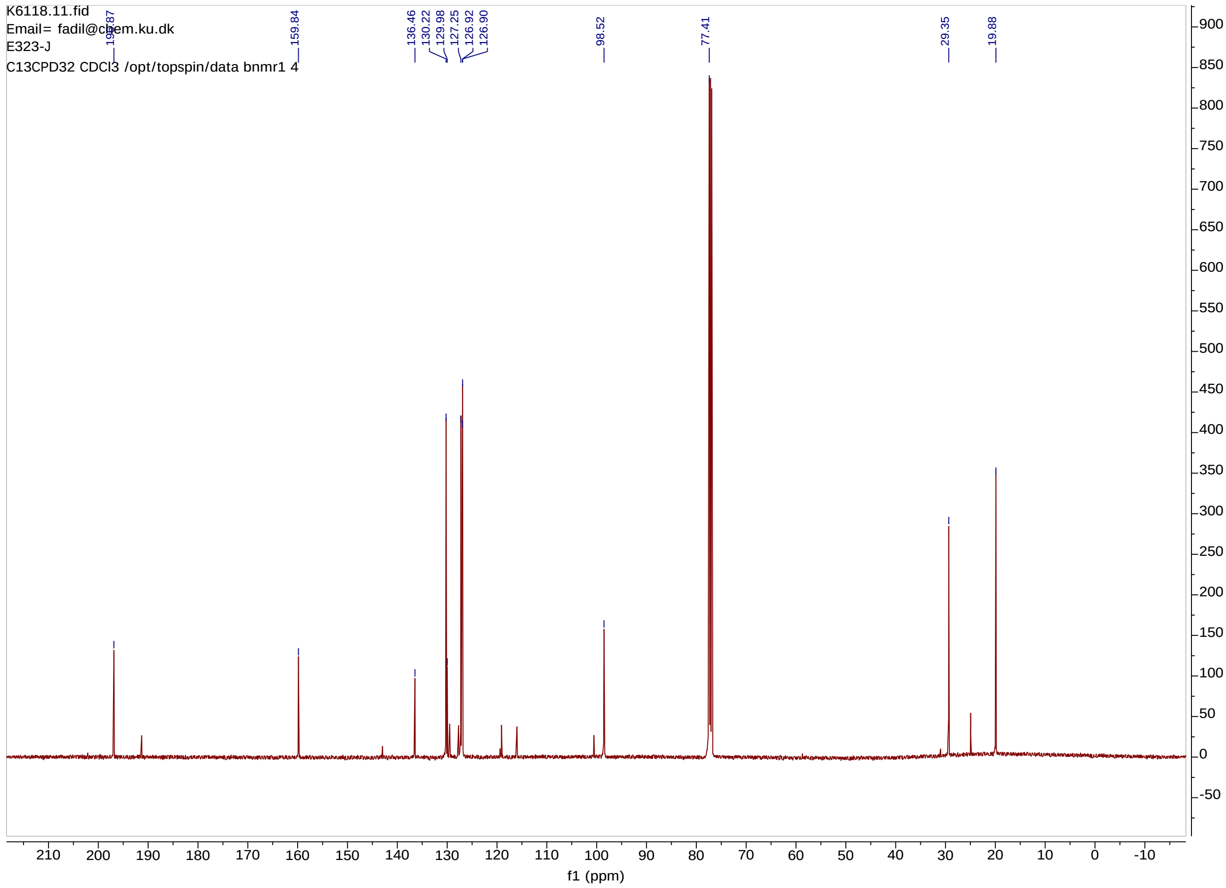

^{13}C NMR spectrum of compound **10**

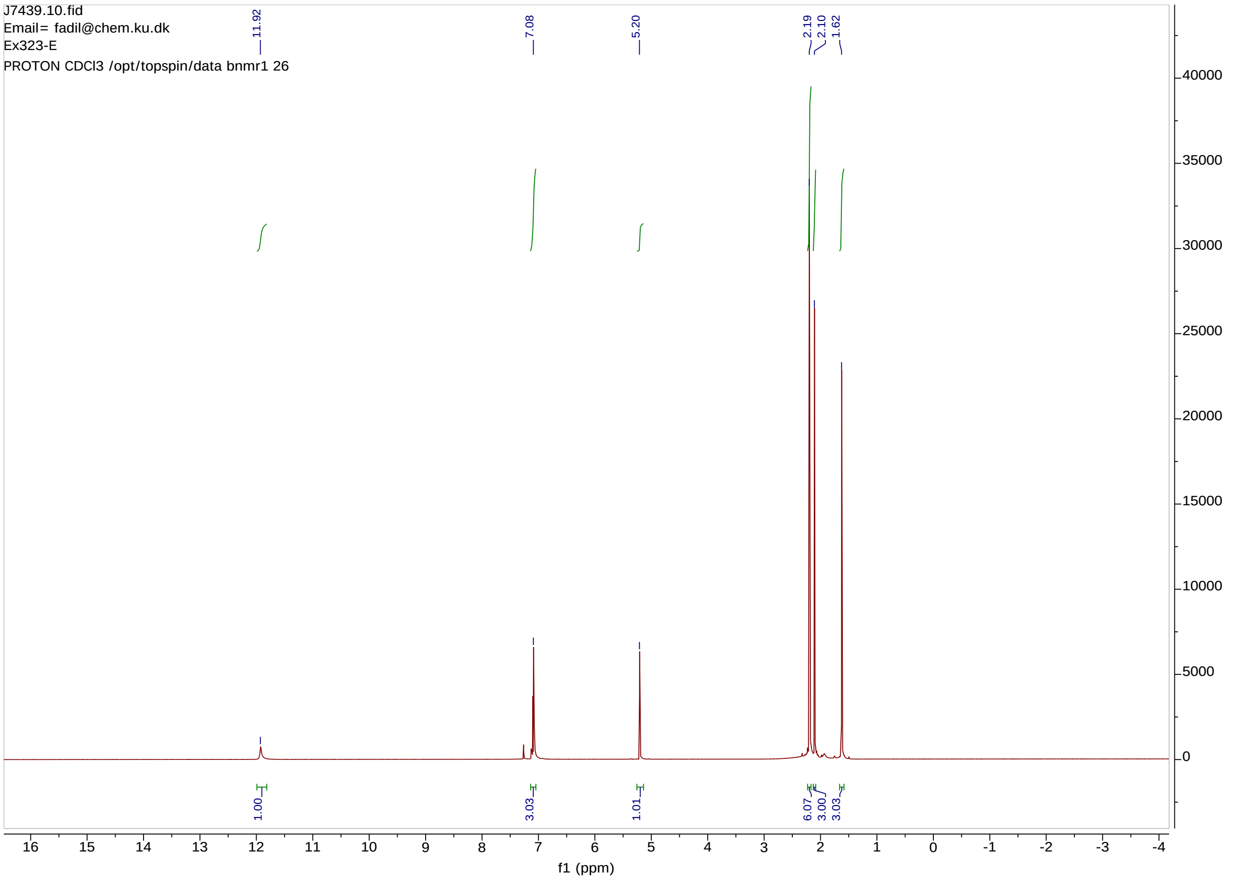

^{1}H NMR spectrum of compound **11**

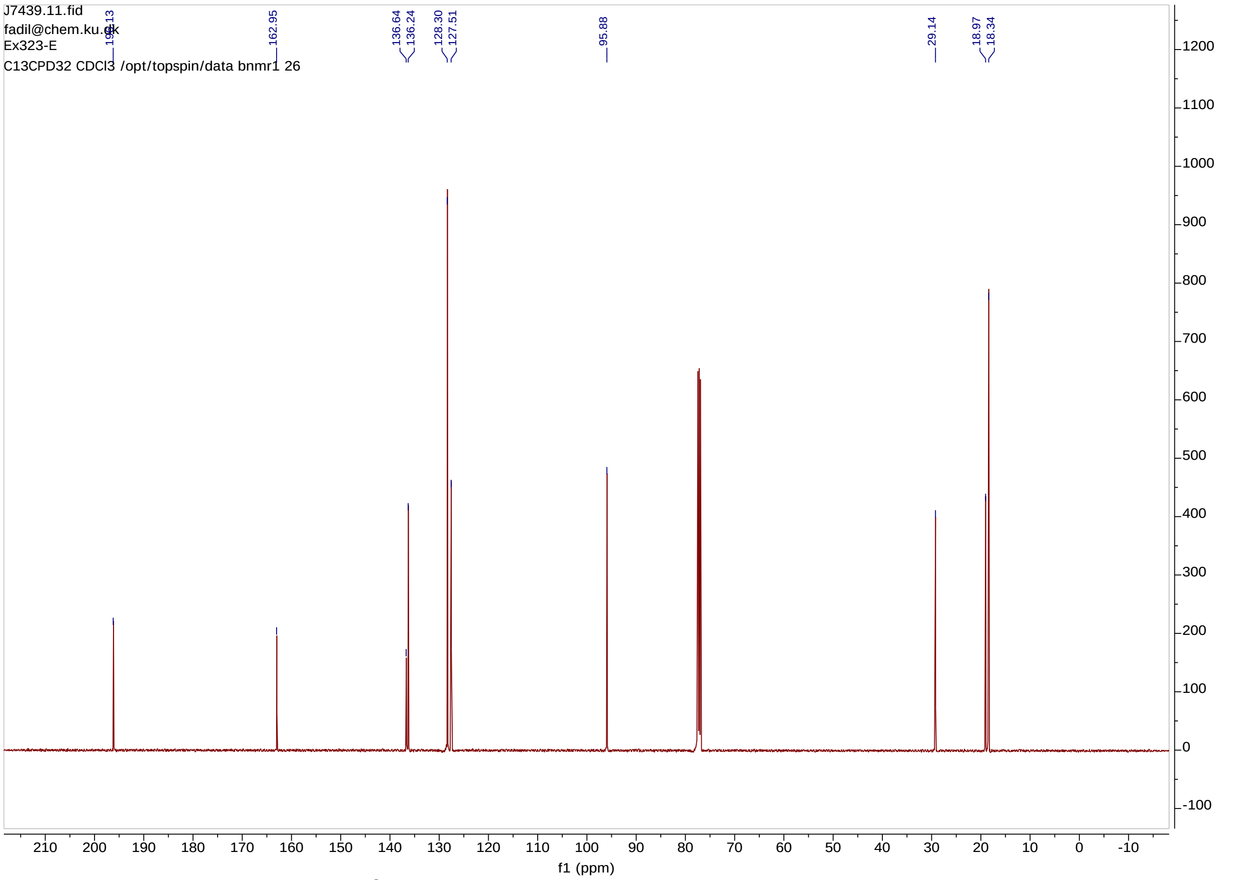

^{13}C NMR spectrum of compound **11**

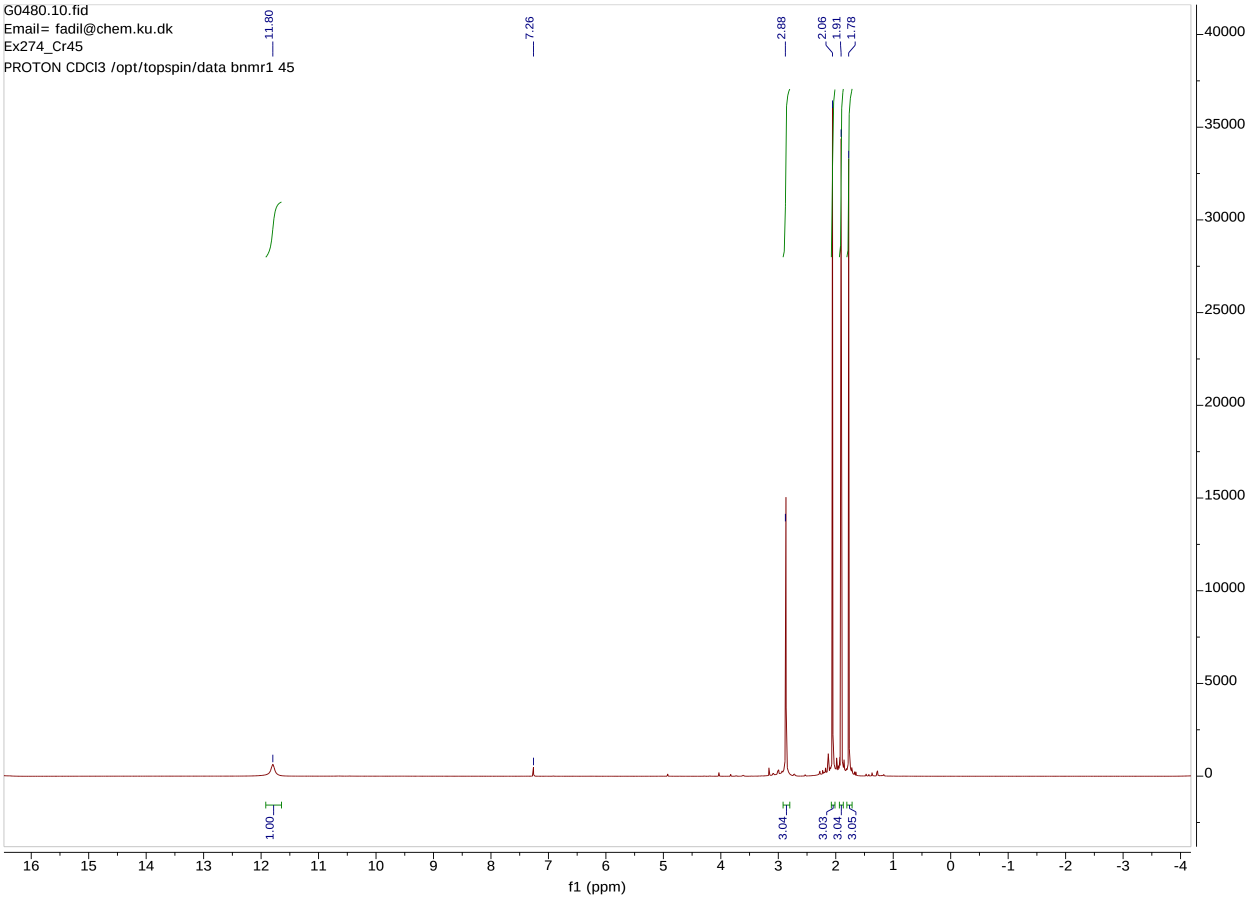

^{1}H NMR spectrum of compound **14**

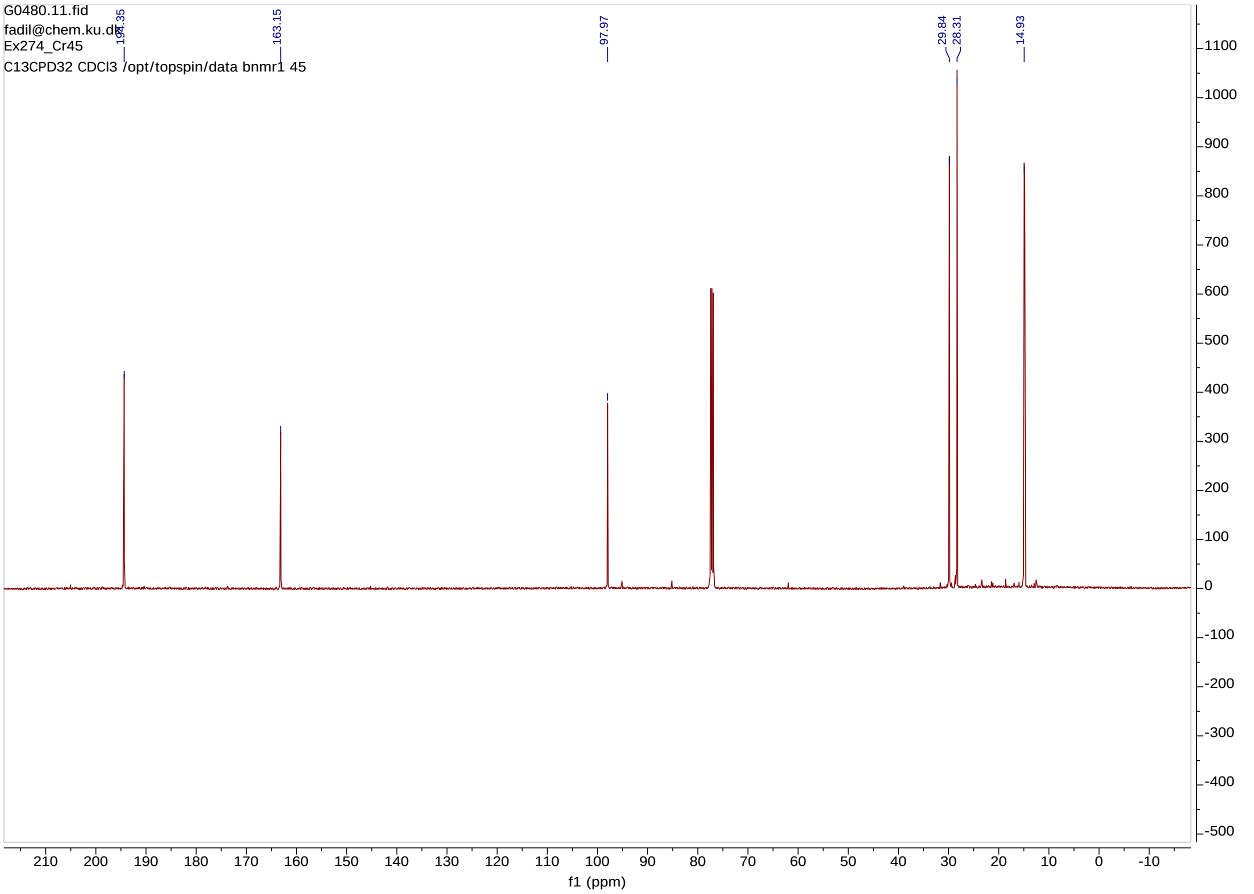

^{13}C NMR spectrum of compound **14**

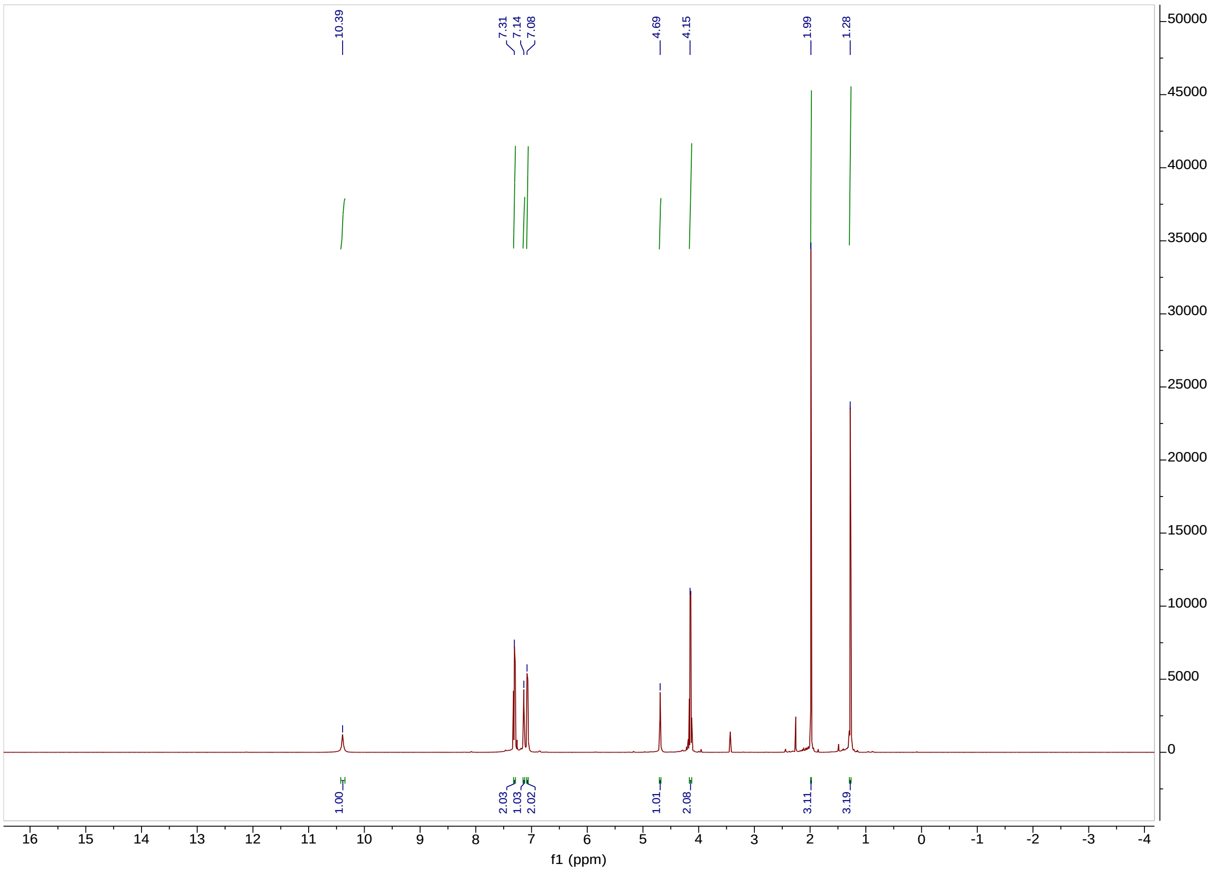

^{1}H NMR spectrum of compound **16**

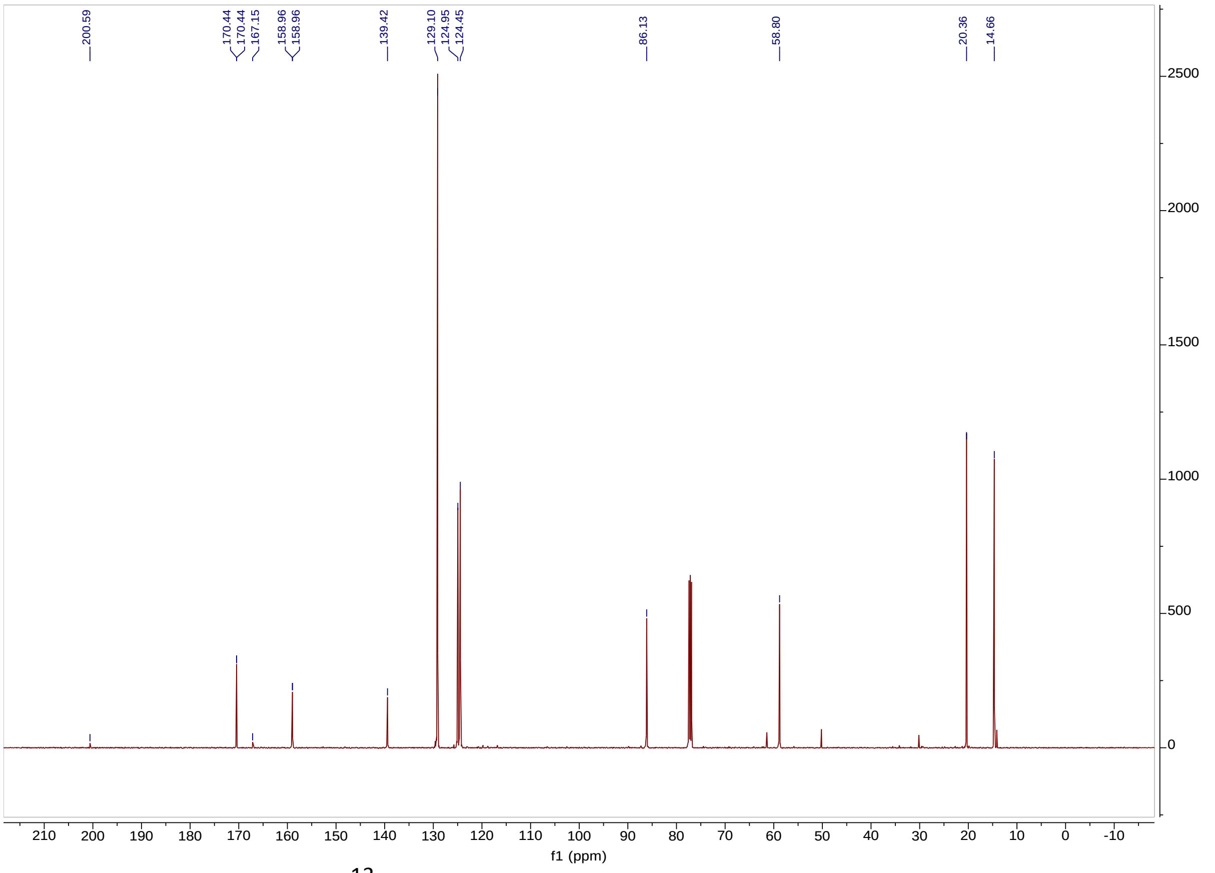

^{13}C NMR spectrum of compound **16**

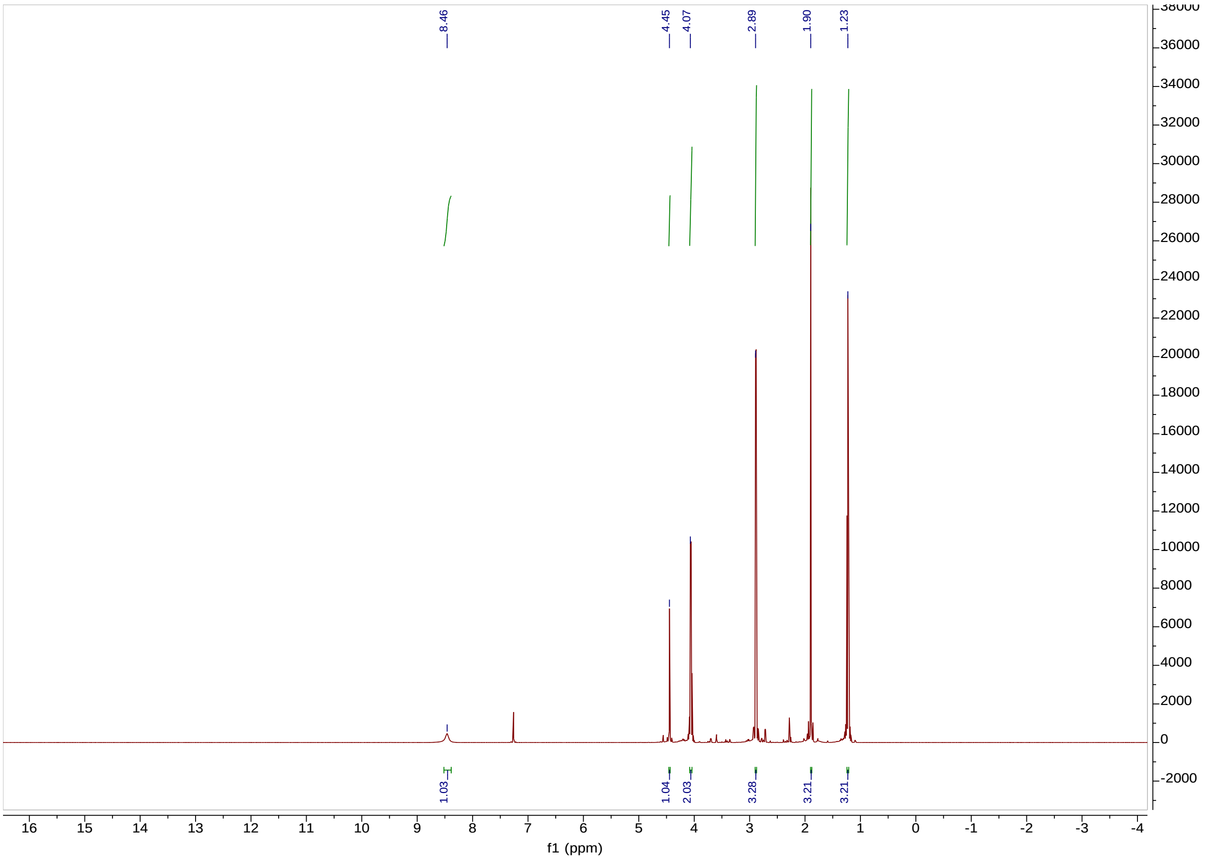

^1H NMR spectrum of compound **17**

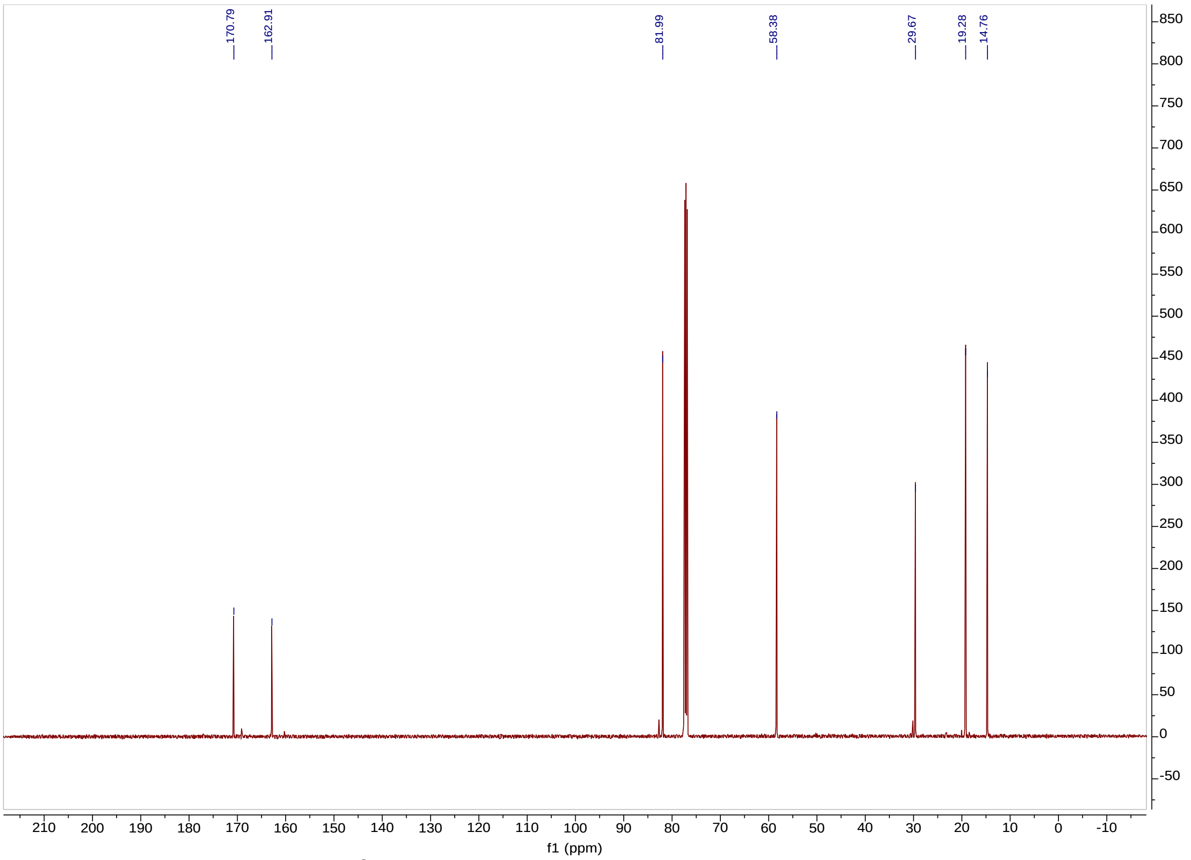

^{13}C NMR spectrum of compound **17**

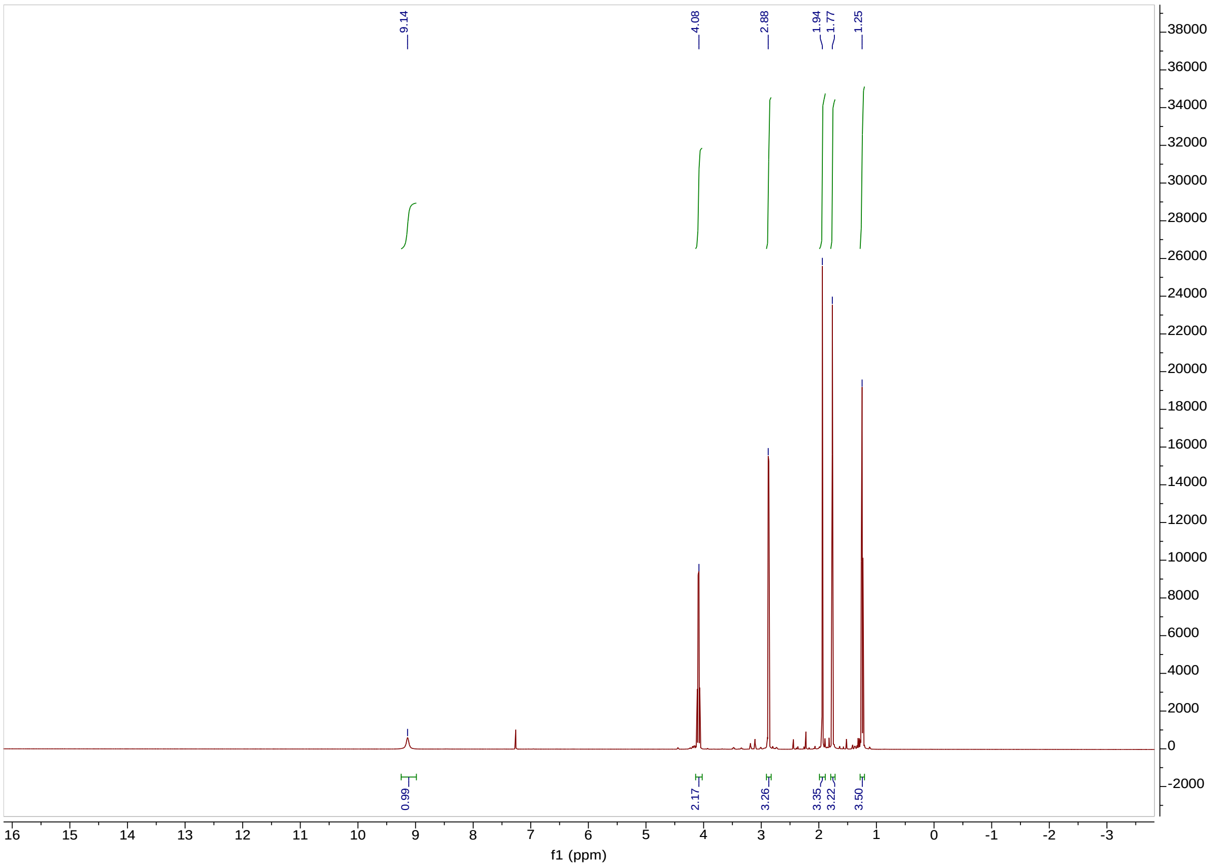

^{1}H NMR spectrum of compound **19**

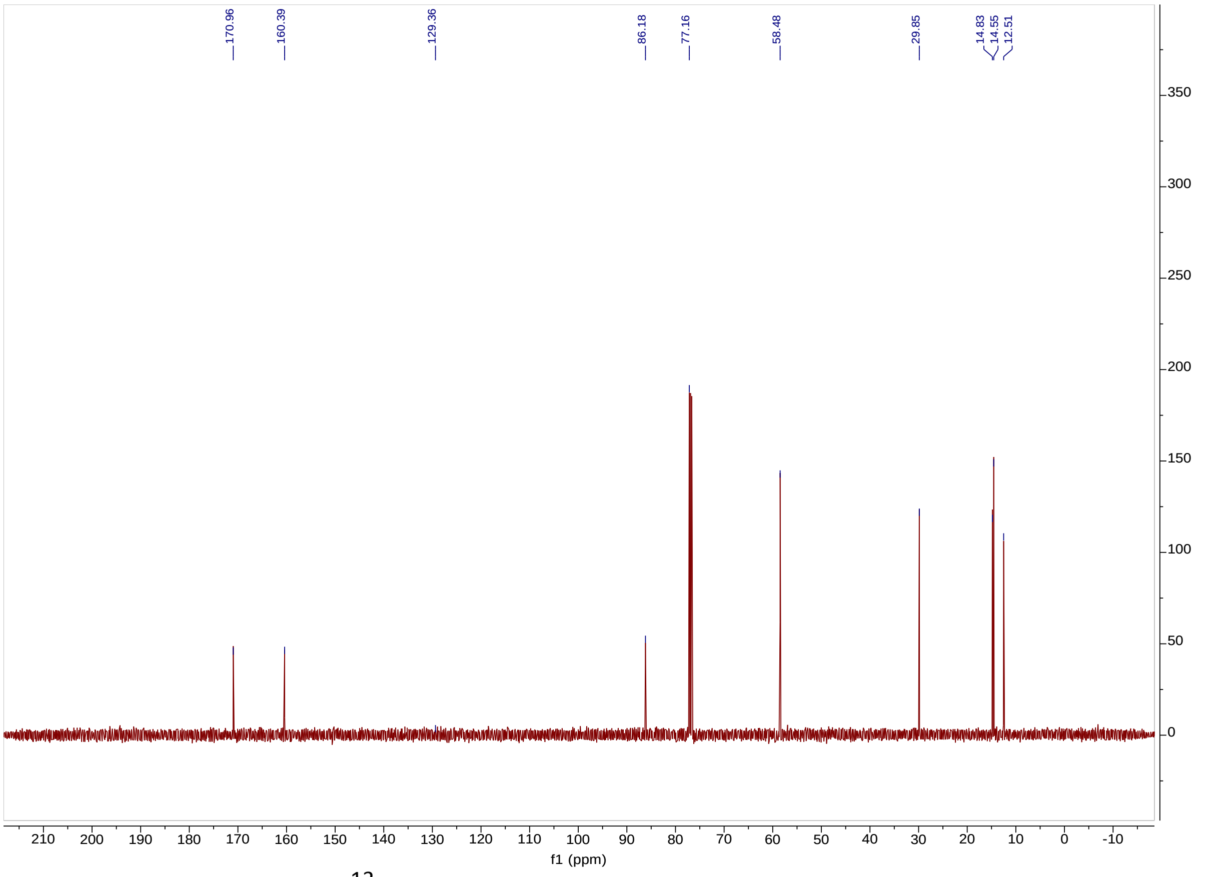

^{13}C NMR spectrum of compound **19**

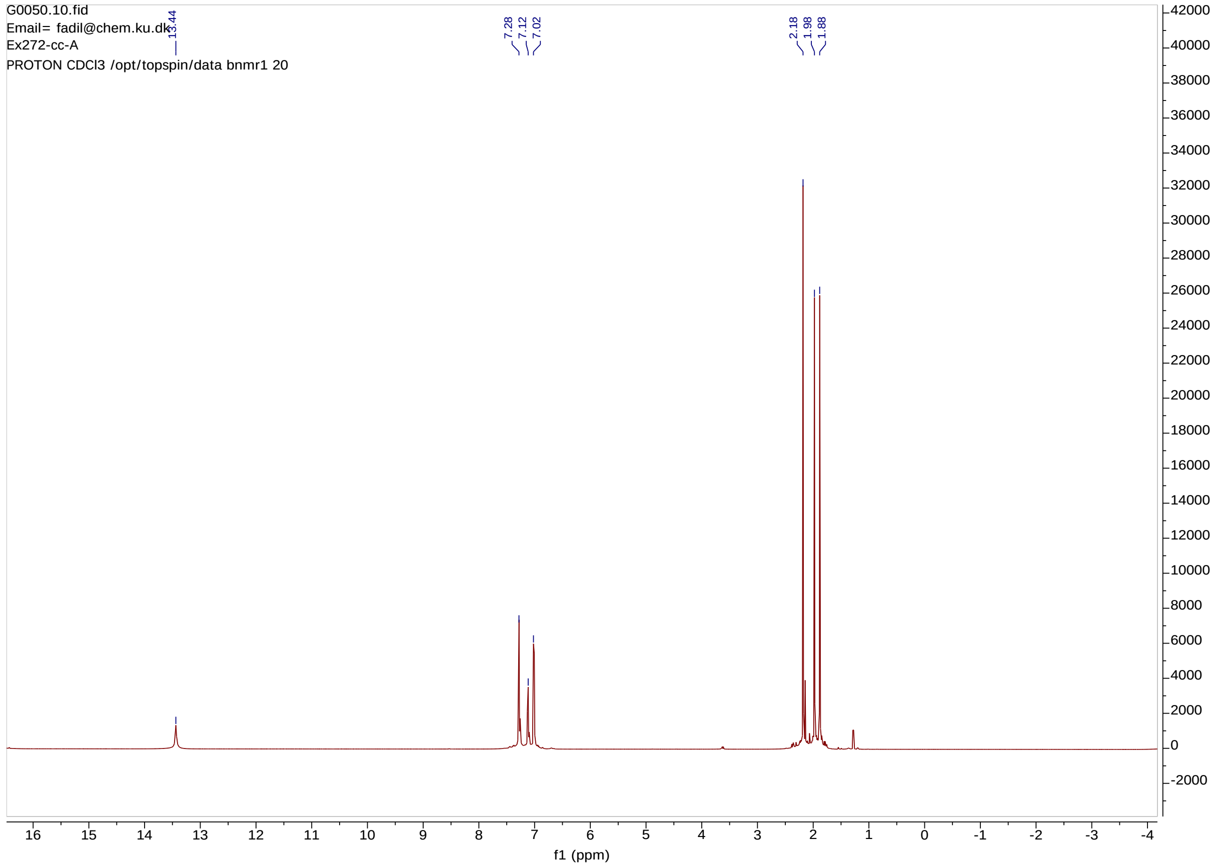

^{1}H NMR spectrum of compound **45**

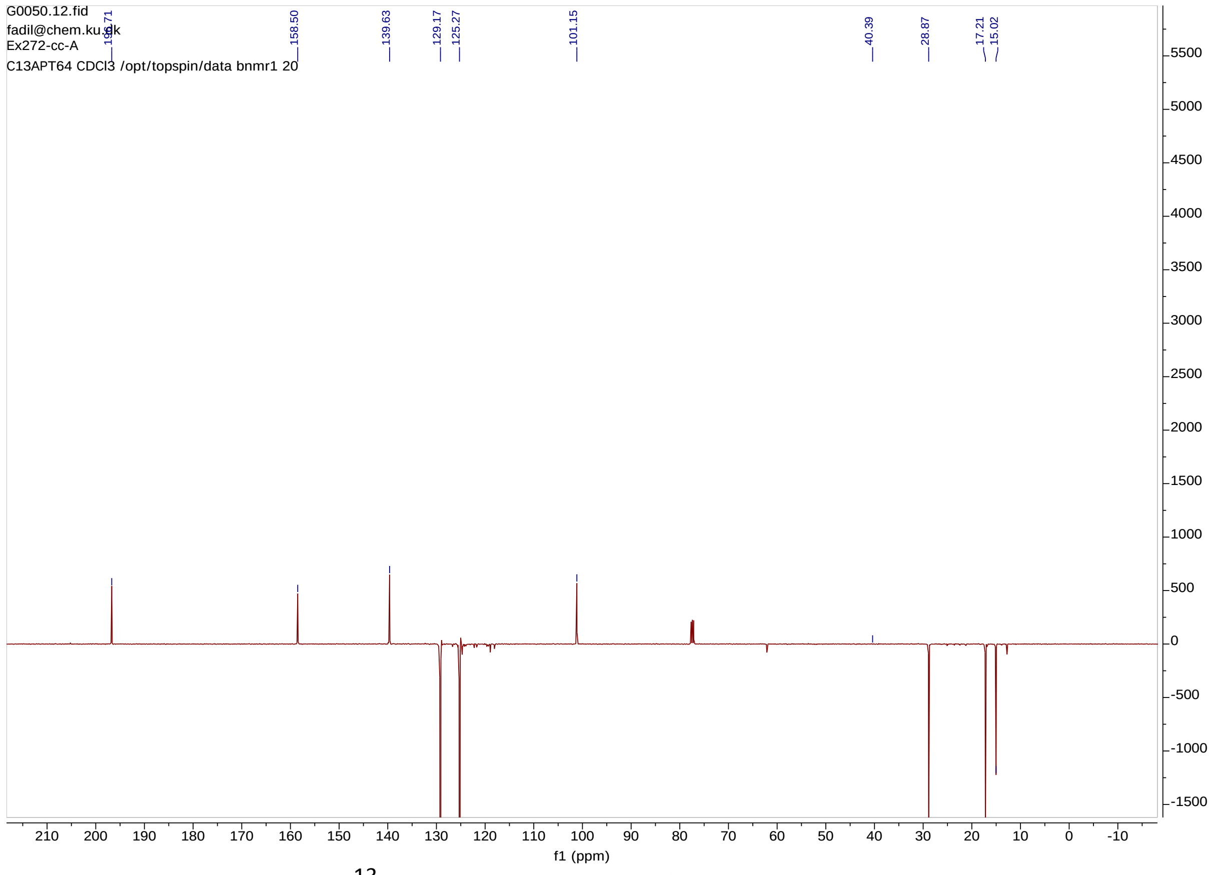

^{13}C NMR spectrum of compound **45**
